# Spatial and temporal plasticity of chromatin during programmed DNA-reorganization in *Stylonychia *macronuclear development

**DOI:** 10.1186/1756-8935-1-3

**Published:** 2008-10-27

**Authors:** Jan Postberg, Katharina Heyse, Marion Cremer, Thomas Cremer, Hans J Lipps

**Affiliations:** 1 Institute of Cell Biology, University Witten/Herdecke, Witten, Germany; 2 Department Biology II, Anthropology and Human Genetics, LMU Munich, Martinsried, Germany; 3 Center for Integrated Protein Science Munich (CIPSM), LMU Munich, Munich, Germany

## Abstract

**Background:**

In this study we exploit the unique genome organization of ciliates to characterize the biological function of histone modification patterns and chromatin plasticity for the processing of specific DNA sequences during a nuclear differentiation process. Ciliates are single-cell eukaryotes containing two morphologically and functionally specialized types of nuclei, the somatic macronucleus and the germline micronucleus. In the course of sexual reproduction a new macronucleus develops from a micronuclear derivative. During this process specific DNA sequences are eliminated from the genome, while sequences that will be transcribed in the mature macronucleus are retained.

**Results:**

We show by immunofluorescence microscopy, Western analyses and chromatin immunoprecipitation (ChIP) experiments that each nuclear type establishes its specific histone modification signature. Our analyses reveal that the early macronuclear anlage adopts a permissive chromatin state immediately after the fusion of two heterochromatic germline micronuclei. As macronuclear development progresses, repressive histone modifications that specify sequences to be eliminated are introduced *de novo*. ChIP analyses demonstrate that permissive histone modifications are associated with sequences that will be retained in the new macronucleus. Furthermore, our data support the hypothesis that a PIWI-family protein is involved in a transnuclear cross-talk and in the RNAi-dependent control of developmental chromatin reorganization.

**Conclusion:**

Based on these data we present a comprehensive analysis of the spatial and temporal pattern of histone modifications during this nuclear differentiation process. Results obtained in this study may also be relevant for our understanding of chromatin plasticity during metazoan embryogenesis.

## Background

It is well established that specific post-translational modifications of histone tails create chromatin structures favorable either for activation or repression of genes. Histone modifications occur in a dynamic fashion and numerous enzymes that introduce histone modifications have been identified, as well as various enzymes that specifically remove these modifications. Such enzymes include histone acetylases and deacetylases, histone methyltransferases and histone demethylases [1-5]. Recently, evidence emerged that small RNAs can act as determinants to recruit chromatin-modifying enzymes, and thus control histone modifications [6-11], but a detailed characterization of such pathways in various organisms is still missing.

Ciliated protozoa represent a unique biological system to analyze the relevance of specific histone modifications for both gene activation and repression and for the specific genome reorganization during a nuclear differentiation process. Each ciliate cell contains two different types of nuclei, one or more macronuclei and one or more diploid micronuclei [12]. All transcripts required for vegetative growth are derived from macronuclear DNA while the DNA in the micronucleus is organized into heterochromatin and is transcriptionally inert. The main function of the micronucleus becomes obvious only during sexual reproduction, the conjugation, which can be induced by mixing ciliate cells of different mating types. Although there is some variation between different ciliate species in the events occurring during conjugation, they all share the same common principle. The micronucleus undergoes a meiotic division and all except one of the resulting daughter nuclei disintegrate. This haploid nucleus then divides mitotically. One of the two resulting nuclei migrates to the conjugation partner, fusing there with the respective haploid counterpart to form a diploid zygote nucleus. This nucleus undergoes mitosis and one of the daughter nuclei differentiates into the transcriptionally inert micronucleus, while the other (the macronuclear anlage) develops into a new transcriptionally active macronucleus [12-14].

The most extreme DNA reorganization events are observed in spirotrichous ciliates, such as *Euplotes*, *Oxytricha *or *Stylonychia*, making them attractive model systems to study the structural and molecular events occurring during nuclear differentiation. In these species the first rounds of DNA amplification lead to the formation of polytene chromosomes with a 100- to 200-fold polyteny. These polytene chromosomes consecutively become degraded and over 90% of micronuclear DNA is eliminated from the macronuclear anlage [13]. Finally, DNA fragmentation leads to short DNA molecules referred to as 'nanochromosomes' [15] with sizes between about 0.4 and 20 kbp, which in general encode only one open reading frame [12,14].

**Figure 1:**
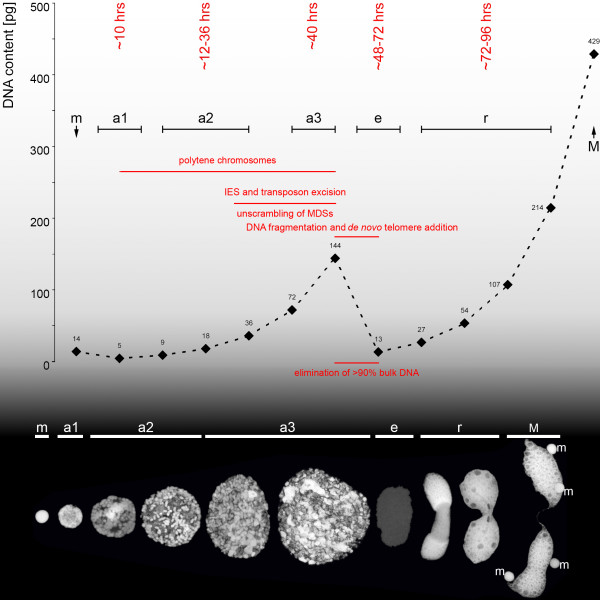
**DNA content and morphology of macronuclear developmental stages**. DNA content values were taken from [13]. After separation of the conjugation partners, the macronuclear anlage at an early stage (a_1_) is only slightly larger than a micronucleus (m). At this stage the beginning of chromatin decompaction is observed. As macronuclear development progresses (a_2_) spiralized chromosomes become visible, which subsequently develop into polytene giant chromosomes (a_3_) during the first rounds of DNA amplification. In this stage excision of internal eliminated sequences, unscrambling of macronucleus destined sequences of scrambled genes and excision of transposon-like elements occur. At subsequent DNA elimination stages (e) bulk micronuclear sequences are removed resulting in a DNA-poor stage containing less than 10% of micronuclear sequences. Further rounds of DNA amplification then take place (r), resulting in a mature nanochromosomes-containing macronucleus (M). For details see [12-14].

Figure 1 gives an overview of the changes in DNA content and illustrates morphological features characteristic of the various macronuclear developmental stages of the ciliate *Stylonychia*. The first rounds of DNA amplification (stages a_1–3_) lead to the formation of polytene chromosomes while coevally a subset of DNA sequences become excised. These include transposon-like elements and internal eliminated sequences (IES), which interrupt macronucleus destined sequences (MDS) [14]. Subsequently polytene chromosomes become degraded (stage a_3_) and a DNA fragmentation process takes place, accompanied by the elimination of bulk micronucleus-specific DNA sequences resulting in a 'DNA poor' stage (stage e). Telomeres are added *de novo *to the remaining DNA molecules. Finally, further rounds of DNA amplification (stage r) result in the formation of the vegetative macronucleus (stage M), in which each individual nanochromosome is present in a specific copy number [12,14].

Early microscopic analyses demonstrated that during polytene chromosome formation extensive chromatin reorganization processes take place. When spread on a water surface, chromatin of the micronucleus appears as coiled 30 nm fibers interconnected by short 10 nm fibers. In the course of polytene chromosome formation 30 nm fibers become organized into loop-like structures. The DNA content of these loops varies between 10 to 200 kbp DNA, very similar to the DNA content of polytene chromosome bands in Diptera [16]. During polytene chromosome degradation the 30 nm chromatin loops are released from the axis and degraded while the 10 nm fibers are retained. These observations suggest that sequences to be excised and eliminated become organized into more compact chromatin structures, while the MDSs adopt a more accessible chromatin structure [16,17]. More recently, direct evidence was provided that an RNAi-dependent mechanism regulates the specific excision and elimination of micronucleus-specific DNA sequences [7,8,18]. Although this mechanism is not yet understood in detail, it seems that small RNA molecules (referred to as scanRNAs in the ciliate *Tetrahymena thermophila*) result from bi-directional transcription of the micronuclear genome during conjugation [19]. One of three Dicer-homologous proteins identified in *Tetrahymena *was shown to be involved in macronuclear development, thus being a candidate for processing of scanRNAs [20]. In *Tetrahymena *experimental evidence has been provided that a protein of the PIWI-family, Twi1p, is required for scanRNA trafficking between the different types of nuclei [7]. Twi1p is thought to direct small RNAs first into the parental macronucleus where selection of scanRNAs either on the DNA or RNA level occurs. This selection results in an accumulation of Twi1p-bound scanRNAs homologous to micronucleus-specific DNA sequences. These scanRNAs then invade the developing macronucleus, specify complementary sequences to be eliminated, and recruit chromatin-modifying enzymes which introduce H3K27me3 [21] and H3K9me3 [18,22]*de novo*.

Small RNAs were also detected in *Stylonychia *during early stages of macronuclear development [18]. These RNAs are complementary to micronucleus-specific sequences and are similar in size (approximately 25 to 30 nucleotides) to Piwi-interacting RNAs (piRNAs), a germline-specific class of small RNA which occurs in mammalian testes [23,24]. Furthermore, the Piwi-family protein mdp1 was shown to be differentially expressed during macronuclear development [25,26]. Silencing of mdp1 expression by RNAi resulted in severe disturbance of macronuclear development [27]. Using antibodies targeted to H3K9me3, it was also shown that this modification occurred in macronuclear anlagen. In addition, chromatin immunoprecipitation (ChIP) demonstrated that micronucleus-specific sequences are decorated with this hallmark [18]. Treatment of exconjugant cells with the histone deacetylase inhibitor trichostatin A resulted in developmental arrest and breakdown of macronuclear anlagen. Since deacetylation of H3 lysines 9 and 14 is a prerequisite for *de novo *H3 lysine 9 methylation [2], this experiment suggests that heterochromatin formation via H3K9me3 plays an important role in the programmed elimination of DNA in *Stylonychia*.

Although the occurrence and biological relevance of several histone modifications have been studied in various ciliate species [18,21,22,28-32] a comprehensive analysis of histone modifications occurring in the course of macronuclear development is still missing. To gain detailed insights into chromatin plasticity during a nuclear differentiation process we studied the spatial and temporal patterns of a large variety of histone modifications, chromatin-modifying proteins and a protein involved in the RNAi dependent heterochromatin formation during various stages of macronuclear differentiation.

## Results

For our comprehensive analyses of chromatin dynamics in the spirotrichous ciliate *Stylonychia lemnae *we performed *in situ *antibody staining combined with confocal laser scanning microscopy (CLSM), Western analyses and in selected cases chromatin immunoprecipitation (ChIP) analyses of chromatin isolated from advanced stages (a_2–3_) of macronuclear anlagen. For details on the different stages of macronuclear differentiation see Figure 1. An overview of all the hallmarks examined and their occurrence in the different nuclear types is provided in Table 1. The specificity of all antibodies used in this study was verified by peptide competition assays (Additional file 1).

**Table 1:** Overview about all histone modifications and proteins examined and their nuclear localization in vegetative and differentiating cells.

	***Vegetative cells***		***Differentiating cells***				
*++ = high level* *+ = moderate level* *- = not detected*	*Micronuclei*	*Macronuclei*	*Micronuclei*	*Parental macronuclei*	*Macronuclear anlagen (a*_1 _*to early a*_2)_	*Macronuclear anlagen (a*_2_*to a*_3_)	*Macronuclear anlagen (e)*

***Histone acetylation***							
H3K9ac	**-**	**++**	**-**	**+^4^**	**++**	**++**	**++**
H3K14ac	**-**	**++**	**-**	**+^4^**	**++**	**++**	**++**
H3K36ac	**-**	**-**	**-**	**-**	**-**	**++**	**+^4^**
***Histone methylation***							
H3K4me1	**-**	**++**	**-**	**+^4^**	**-**	**++**	**++**
H3K4me3	**-**	**++**	**-**	**+^4^**	**-**	**++**	**++**
H3K9me3	**-**	**-**	**-**	**+^5^**	**-**	**++**	**+^4^**
H3K27me1	**-**	**-**	**- (+^1^)**	**-**	**-**	**++**	**+^4^**
H3K27me3	**++**	**-**	**++**	**-**	**-**	**++**	**+^4^**
H3K36me3	**+**	**-**	**+(-^2^)**	**-**	**-**	**+**	**+^4^**
***Histone phosphorylation***							
H3S28p	**++**	**+**	**-**	**+^4^**	**++**	**++**	**++**
***Chromatin modifying proteins***							
E(z)/KMT6-like HMT	**-**	**-**	**-**	**-**	**-**	**++**	**+^4^**
Spdd1p	**-**	**-**	**-**	**+^5^**	**-**	**++**	**+^4^**
***RNAi-related machinery***							
Piwi/mdp1	**-**	**-**	**+^3^**	**++^6^**	**++**	**+^4^**	**+^4^**

^1^transient accumulation during conjugation

^2^transient loss during conjugation

^3^transient accumulation early during conjugation

^4^signal intensity and frequency continuously decreasing

^5^modification occasionally observed as dispersed foci-like signals mostly not visible in stack projections

^6^resrtricted to conjugation and early exconjugant cells with no visible macronuclear anlagen

### The histone signature of nuclei in vegetative *Stylonychia *cells

#### Acetylated histone H3 as well as H3K4me1 and H3K4me3 are abundant in the transcriptionally active macronucleus

**Figure 2:**
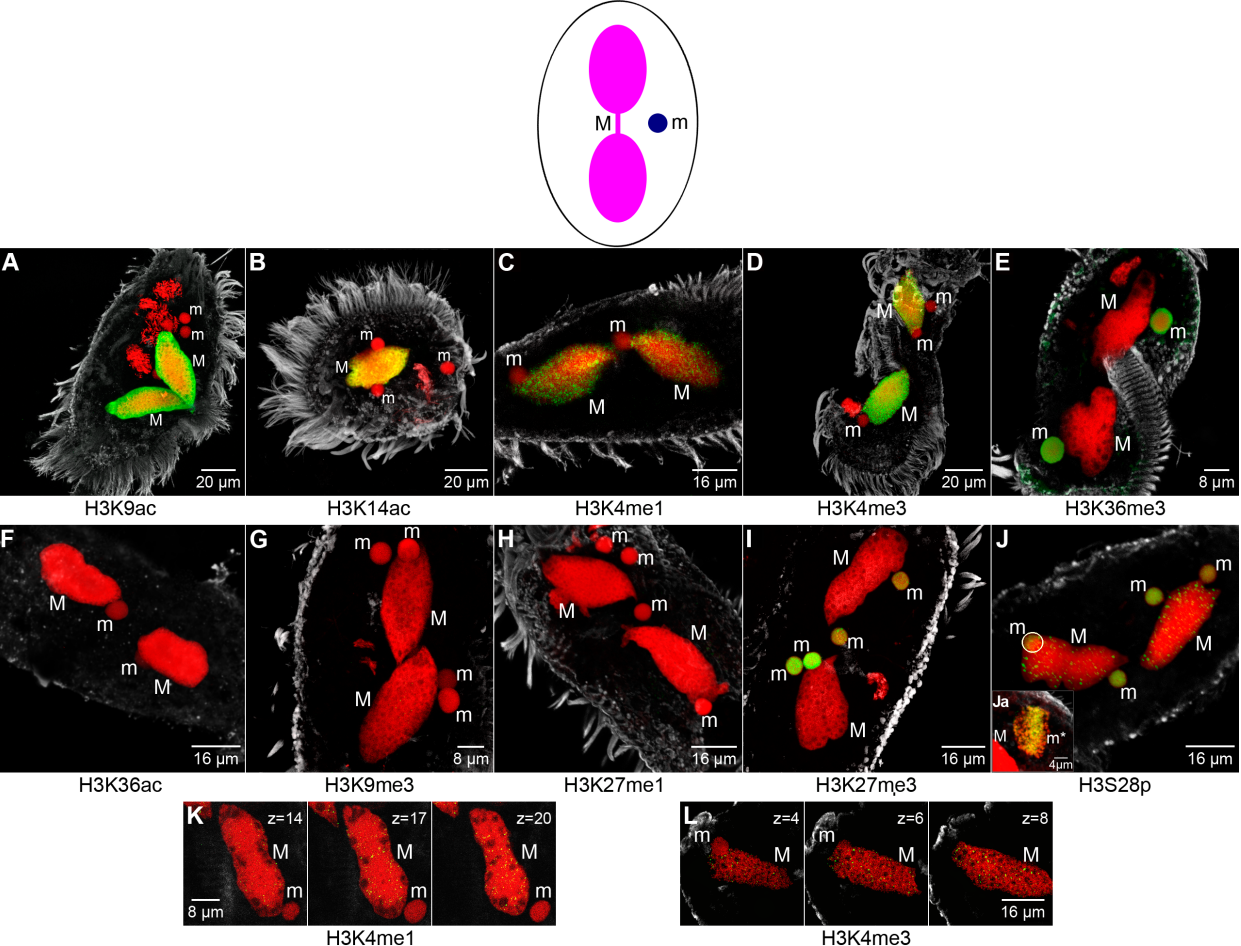
**The histone signature of nuclei in the vegetative cell**. Nuclear types occurring in vegetative *Stylonychia*: the transcriptionally active macronucleus (M) consisting of two ovoid distal parts connected by a thin nucleoplasmic bridge, and the transcriptionally inert micronucleus (m). *In situ *antibody staining of histone H3 modifications (green) and DNA counterstaining (red). The cellular shape was visualized using an α-tubulin-antibody (grey). **(A-J) **Confocal image stack projections. **(K, L) **Mid light optical sections. Histone modifications detected in the macronucleus (M) are H3K9ac **(A)**, H3K14ac **(B)**, H3K4me1 **(C)**, H3K4me3 **(D)**, and H3S28p **(J)**. Histone modifications found in the micronucleus of vegetative *Stylonychia *are H3K36me3 **(E)**, H3K27me3 **(I)**, and H3S28p **(J)**, which is also a prominent micronuclear mitosis (m*) marker **(Ja)**. H3K36ac **(F)**, H3K9me3 **(G) **as well as H3K27me1 **(H) **are not detected in nuclei of vegetative cells.

It is well established that acetylation of histone N-termini is necessary to transform or maintain chromatin in a transcriptionally competent state in many organisms [2]. Using specific antibodies the presence of H3K9ac (Figure 2A) and H3K14ac (Figure 2B) in macronuclei (M) and the absence of both modifications in micronuclei (m) was demonstrated by *in situ *antibody staining. Western analyses confirmed that H3K9ac/K14ac was abundant in macronuclei, but could not be detected in micronuclei (Additional file 2A). Notably, using *in situ *antibody staining we could not observe H3K36ac in macronuclei (M) or micronuclei (m). The existence of this modification has recently been demonstrated in *Tetrahymena*, *Saccharomyces cerevisiae *and human [33].

H3K4 methylation has been implicated in gene activation as well as in gene silencing [34]. H3K4me1 is a frequent histone modification in macronuclei of *Tetrahymena *as well as in nuclei of *S. cerevisiae *and human [29,32]. This modification has been linked to silenced euchromatin in *Chlamydomonas *[35]. H3K4me3 is found to be associated with 5' ends of euchromatic genes, which contain RNA-Pol II initiation sites [36-38]. It has been suggested that H3K4me3 is related to the process of active transcription, even though a very recent genome-wide study showed that this modification is found at most promoters of protein-coding genes in human cells, regardless of whether they produce full-length transcripts or not [39]. Using immunofluorescence microscopy H3K4me1 (Figure 2C) could not be detected in micronuclei (m) but occurred as foci-like signals in the macronucleus (M). Similar to H3K4me1, H3K4me3 (Figure 2D) was a prominent marker in macronuclei (M), but was also not detected in micronuclei (m). When DNA is stained with To-Pro-3, the macronucleus reveals a sponge-like structure with domains of condensed chromatin separated from regions almost devoid of chromatin [40]. It has been shown that nascent Pol I and Pol II transcripts occur at borders between such chromatin-rich and chromatin-poor compartments [41]. While the preferential localization of H3K4me1 (Figure 2K) at such compartment borders was not observed in single light optical sections, H3K4me3 (Figure 2L) exhibited a similar distribution as described earlier for nascent RNA. This observation suggests that H3K4me3 is associated with active transcription in macronuclei. The abundance of H3K4me1 as well as of H3K4me3 in macronuclei and the absence of both modifications in micronuclei was confirmed by Western analyses (Additional file 2A).

#### H3K36me3 and H3K27me3 characterize the germline micronucleus

H3K36me3 has been reported to accumulate at the 3' end of transcriptionally competent genes and is thought to act as a recruiting platform for histone deacetylase (HDAC) activity, preventing internal initiation of mRNA coding sequences in yeast [42]. In ciliates the sole methylation observed on H3K36 to date is H3K36me1, in purified *Tetrahymena *macronuclei [29,32]. However, in relation to other organisms, for example human, mouse or *Arabidopsis*, the local sequence motif around H3K36 appears to be less conserved in *Tetrahymena *than in most H3 variants of *Stylonychia *[43]. Interestingly, in our study H3K36me3 was found in micronuclei (m), whereas this modification was not observed in transcriptionally active macronuclei (M) by immunofluorescence (Figure 2E) and Western analyses (Additional file 2A).

H3K9me3 is implicated in transcriptional repression of euchromatic genes and to the formation of pericentric heterochromatin [2,4]. Previous analyses using antibodies targeted to repressive lysine methylation sites at histone H3 suggested that H3K9me3 occurred in macronuclear anlagen and in micronuclei of *Stylonychia *[18]. It was later confirmed that the antibody directed against H3K9me3 (Abcam, product code: ab8898) used in [18] cross-reacted with H3K27me3, as documented by the manufacturer. Our immunofluorescence analyses have now clarified that H3K9me3 (Figure 2G) is neither present in micronuclei (m) nor in macronuclei (M).

Similar to H3K9me3, H3K27me1 is highly enriched at pericentric heterochromatin in mammalian cell nuclei [44]. This modification was reported to be abundant in macronuclei of *Tetrahymena *[29,32]. In contrast, in *Stylonychia *H3K27me1 was neither detected in micronuclei (m) nor in macronuclei (M) (Figure 2H).

H3K27me3 has been related to HOX gene silencing, X chromosome inactivation and genomic imprinting. This silencing system is linked to the function of the polycomb group of proteins (PcG) since complexes composed of variable PcG proteins have been shown to catalyze methylation of H3K27 [4,45]. In *Tetrahymena*, H3K27me3 has been detected in both the micronucleus and the macronucleus [21,29,32]. *In situ *antibody staining in our study revealed an abundance of H3K27me3 in micronuclei (m) of vegetative *Stylonychia *(Figure 2I), suggesting that the micronucleus-specific H3 variant 'X' [46] carries this modification. In contrast, H3K27me3 was not detected in macronuclei (M).

During mitosis Ser-10 and Ser-28 of H3 become phosphorylated by the kinase Aurora-B [47,48]. It was shown that H3S10p can occur, even if the neighboring lysine 9 is methylated. H3S10p is sufficient to prevent or disrupt HP1 binding at H3K9me3 [49,50], similarly to H1S27p preventing HP1 binding at H1K26me3 [51]. Such binary switches have been preassigned as 'methyl/phospho-switch' [52]. The effects of phosphorylation at Ser-28 of histone H3 are far less characterized. Recently, this modification has been associated with transcriptional activity in immature chicken erythrocytes [53]. Our immunofluorescence microscopic analyses revealed that H3S28p was abundant in micronuclei (m*) during mitosis (Figure 2Ja), but this modification was also detected in interphase micronuclei (m) of vegetative cells (Figure 2J) as well as in macronuclei (M) as dispersed foci-like signals that were apparently localized at surfaces separating condensed chromatin domains and regions almost devoid of chromatin.

### Chromatin dynamics during macronuclear development

#### The conjugating cell

**Figure 3:**
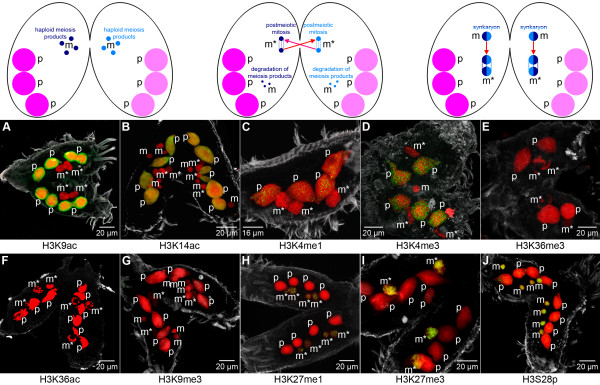
**Histone modifications during conjugation**. Nuclear events induced at the onset of sexual reproduction, when two *Stylonychia *cells of different mating types conjugate. **Left: **The old macronucleus is fragmented into a variable number of fragments (p), which partly persist in subsequent stages of differentiating cells, but are eventually degraded. Meiosis of diploid micronuclei (m) leads to haploid intermediates. **Middle: **One haploid micronucleus in each cell undergoes a postmeiotic mitosis (m*); remaining micronuclei (m) are degraded. One of the resulting haploid nuclei becomes a migratory micronucleus, and these nuclei are exchanged among the conjugating cells. **Right: **The migratory micronuclei fuse with their stationary counterparts to build a diploid synkaryotic micronucleus (m). This synkaryon divides mitotically, and one of the resulting nuclei becomes a new micronucleus. The other differentiates into a new macronucleus. *In situ *antibody staining (color code as described in Figure 2): Similar to the macronucleus of vegetative cells, several histone modifications are detected in the fragmented parental macronucleus (p): H3K9ac **(A)**, H3K14ac **(B)**, H3K4me1 **(C)**, H3K4me3 **(D)**, and H3S28p **(J)**. H3K36me3 is lost from the micronucleus at this stage **(E)**, whereas H3K27me3 **(I)**, and H3S28p **(J) **persist. H3K27me1 **(H) **is transiently detected in micronuclei (m*) exclusively at this developmental stage.

During conjugation the parental macronucleus disintegrates into a variable number of fragments, which are at least partly still detectable in the cell during subsequent stages of macronuclear development. Our immunofluorescence analyses revealed that the histone modification pattern of these fragments does not differ from the pattern of vegetative macronuclei. Strong signals were observed in the parental macronuclei (p) using antibodies directed against H3K9ac (Figure 3A) or H3K14ac (Figure 3B). H3K4me1 (Figure 3C) and H3K4me3 (Figure 3D) also were prominent hallmarks although with weaker signal intensity than that observed in the vegetative macronuclei (M in Figure 2C, D), and H3S28p (Figure 3J) occurred as scattered foci-like signals in the fragmented parental macronuclei (p). These observations suggest that the chromatin of the parental macronuclear fragments still adopts a transcriptional competent or active state in conjugating cells.

Histone modifications in micronuclei of conjugating cells partly deviate from those of vegetative cells. Since H3K36me3 was not detected in micronuclei undergoing meiosis or mitosis (m*) in conjugating cells (Figure 3E), this modification was obviously lost at the onset of sexual reproduction. Remarkably, H3K27me1 was observed transiently during prophase of meiosis or mitosis of micronuclei (m*) during conjugation (Figure 3H).

#### Early macronuclear differentiation is characterized by the introduction of H3K9ac and H3K14ac

**Figure 4:**
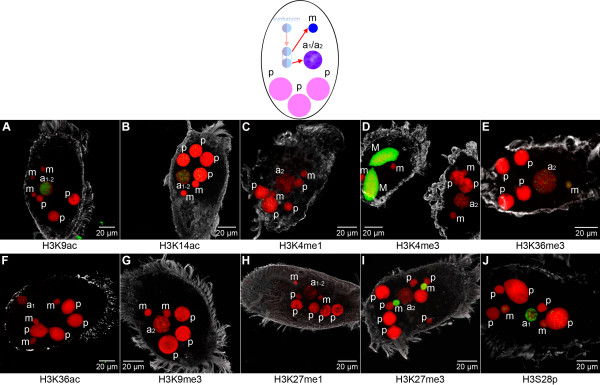
**The histone signature of early developing macronuclear anlagen**. Nuclear types occurring during early macronuclear development: New micronuclei (m) are formed at this stage from one daughter nucleus after mitotic division mitotic division of the zygote nucleus. The other daughter nucleus develops into a macronuclear anlage (a_1_/a_2_), which is characterized by chromatin decompaction, beginning of chromosome polytenization, and an increase in size at this early stage of differentiation. Fragmented parental macronuclei (p) persist at this stage. *In situ *antibody staining (color code as described in Figure 2): Exclusively H3K9ac **(A)**, H3K14ac **(B)**, and H3S28p **(J) **are detected in the very early macronuclear anlagen (a_1_/early a_2_), whereas 'active' chromatin markers H3K4me1 **(C) **and H3K4me3 **(D) **are not observed (a vegetative cell with a prominently stained macronucleus (M) is shown additionally in **(D) **as a positive control). At best, sporadic foci-like signals of H3K36me3 **(E)**, H3K9me3 **(G)**, and H3K27me3 **(I) **are observed at the earliest at stage a_2_. In micronuclei (m) H3K36me3 **(E) **is observed to accumulate *de novo*, whereas H3K27me3 **(I) **persists, and H3S28p **(J) **is lost at this stage.

**Figure 5:**
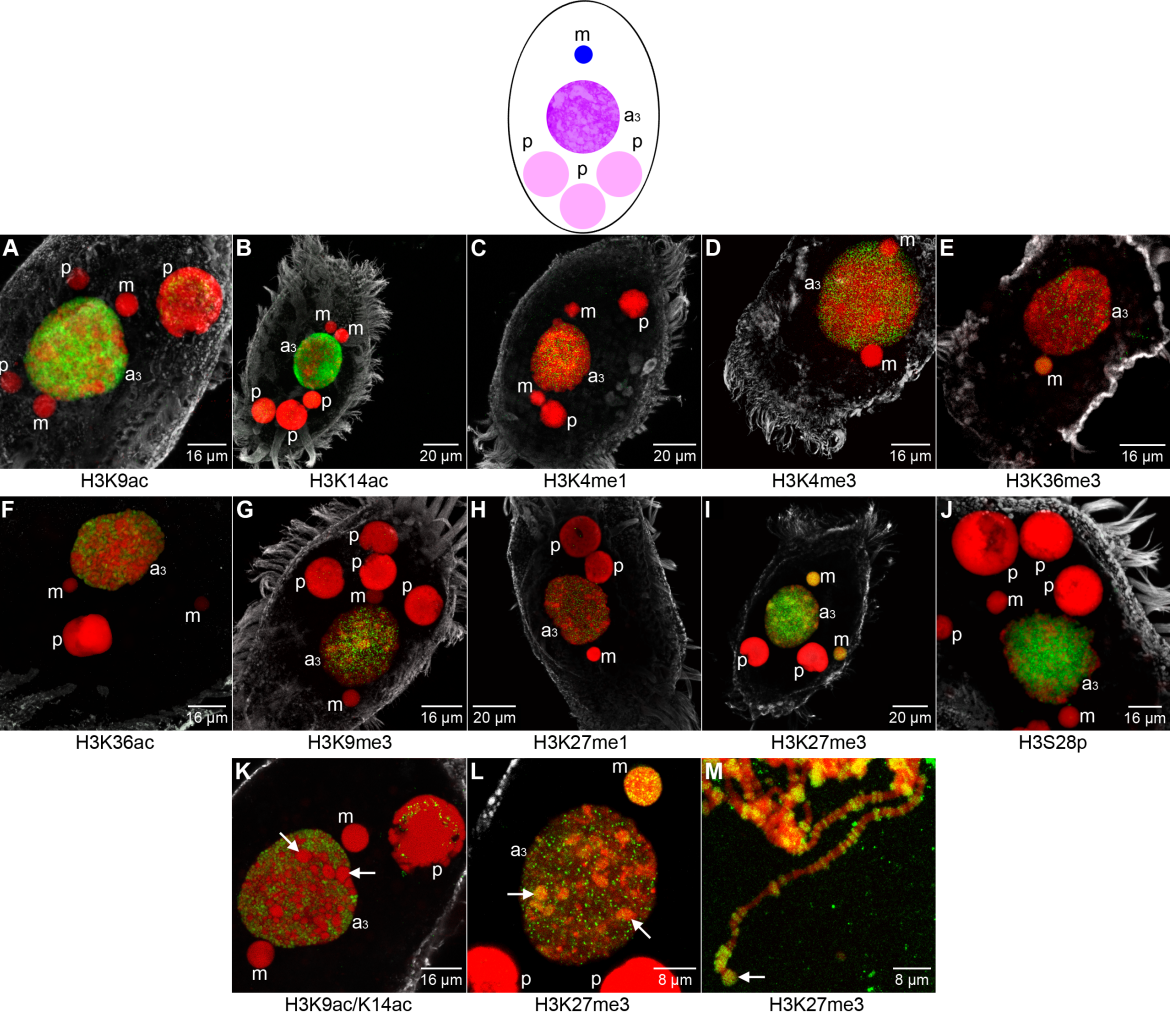
**Histone modifications of advanced macronuclear anlagen with polytene chromosomes**. Nuclear types occurring at advanced developmental stages during the first round of DNA-amplification: Polytene chromosomes are formed in the macronuclear anlage (a_3_). During this stage excision of internal eliminated sequences, unscrambling of macronucleus destined sequences of scrambled genes and excision of transposon-like elements occur. Besides macronuclear anlagen (a_3_), micronuclei (m) and fragmented parental macronuclei (p) are found in the cells. *In situ *antibody staining (color code as described in Figure 2): **(A-J) **Confocal image stack projections. **(K-M) **Mid light optical sections (arrows point at 'heterochromatic blocks'). H3K9ac **(A)**, H3K14ac **(B)**, and H3S28p **(J) **persist with high signal intensity in advanced macronuclear anlagen (a_3_), but H3K9ac/K14ac **(K) **is obviously omitted from large chromatin domains, 'heterochromatic blocks'. H3K4me1 **(C)**, H3K4me3 **(D)**, and H3K36ac **(F) **accumulate in macronuclear anlagen at this stage, and scattered foci-like signals of H3K36me3 **(E) **are also detected. High levels of H3K9me3 **(G)**, H3K27me1 **(H)**, and H3K27me3 **(I) **are introduced *de novo *at stage a_3_. H3K27me3 **(L) **is also detected in large chromatin domains omitted from H3 acetylation. In spread polytene chromosomes H3K27me3 **(M) **is detected in chromosomal bands and 'heterochromatic blocks'.

**Figure 6:**
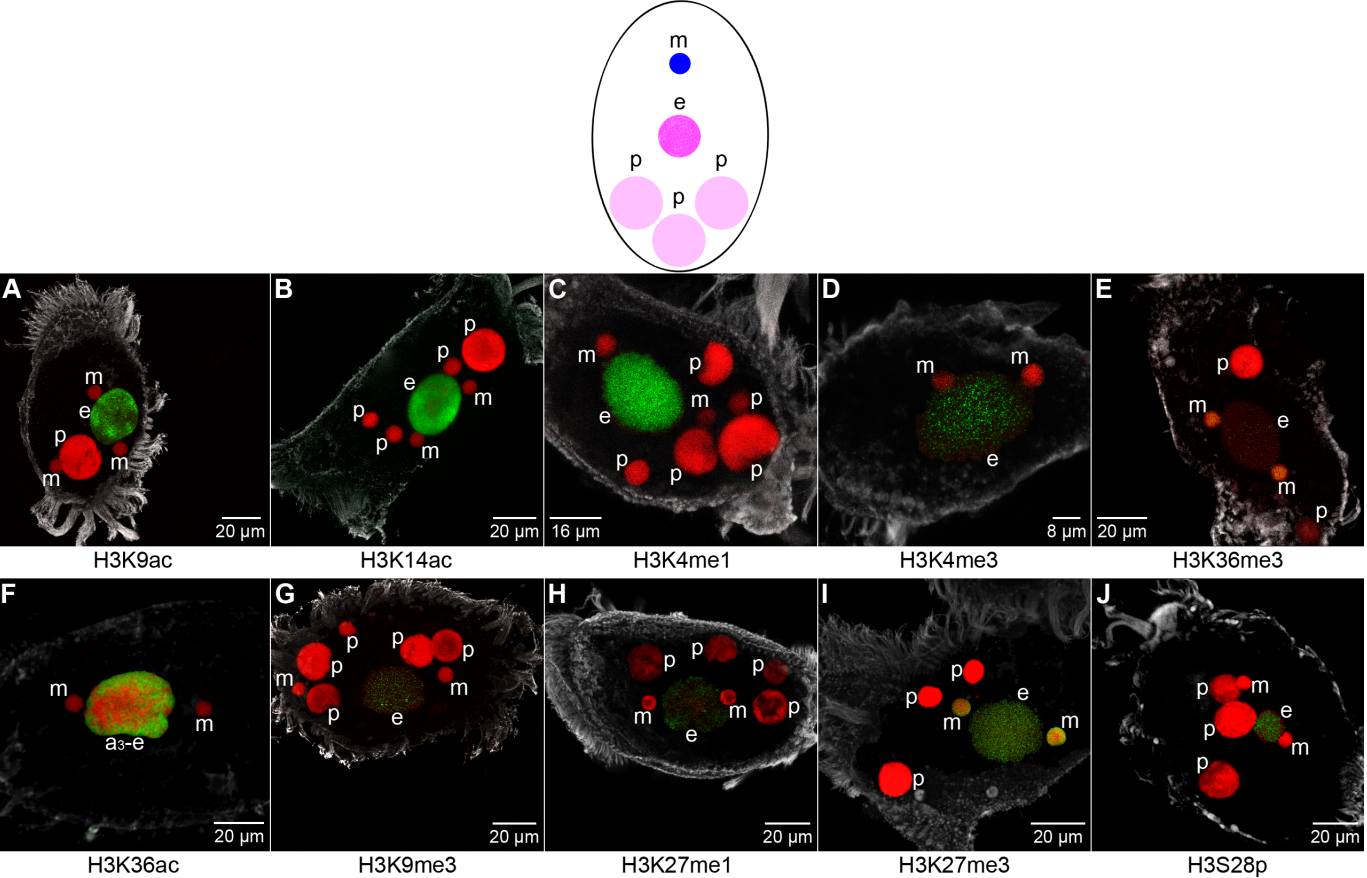
**Histone modifications in macronuclear anlagen during DNA elimination**. Nuclear types occurring during DNA elimination: In macronuclear anlagen (e) at this stage bulk micronuclear sequences are removed resulting in a DNA-poor nucleus containing less than 10% of micronuclear sequences. Further micronuclei (m) and fragmented parental macronuclei (p) are found in the cells. *In situ *antibody staining (color code as described in Figure 2): Histone modifications detected at stage a_3 _of advanced macronuclear anlagen are also detected at stage e during DNA elimination. Importantly, signal intensity of H3K36ac (F) reaches a maximum at the transition from a_3_-e, and signal intensity of H3K36me3 (E), H3K9me3 (F), H3K27me1 (G), and H3K27me3 (H) gradually decreases. Subsequently, all these modifications (E-I) can no longer be detected in prospective macronuclei.

At early developmental stages following the separation of mating cells, the signal intensity of H3K9ac (Figure 4A; Figure 5A; Figure 6A), H3K14ac (Figure 4B; Figure 5B; Figure 6B), H3K4me1 (Figure 4C; Figure 5C; Figure 6C), H3K4me3 (Figure 4D; Figure 5D; Figure 6D) and H3S28p (Figure 4J; Figure 5J; Figure 6J) in the fragmented parental macronuclei (p) gradually became weaker, or in some cases signals were entirely lost. H3K9me3 (Figure 4G; Figure 5G, Figure 6G) and H3K27me1 (Figure 4H; Figure 5H, Figure 6H) occasionally occurred as sporadic foci-like signals in fragmented parental macronuclei (p) of all macronuclear developmental stages.

H3K27me3 was present in micronuclei (m) of exconjugant cells (Figure 4I; Figure 5I; Figure 6I), as it was during conjugation. After the removal of H3K36me3 from micronuclei in the previous developmental stage this modification accumulated *de novo *in micronuclei (m) following the separation of conjugating cells (Figure 4E; Figure 5E; Figure 6E). H3K27me1 (Figure 4H; Figure 5H; Figure 6H) as well as H3S28p (Figure 4J; Figure 5J; Figure 6J) were lost from micronuclei (m) after the separation of mating cells as observed by *in situ *antibody staining, and neither modification was traceable in subsequent developmental stages. Particularly, the loss of H3S28p exhibits a characteristic (epigenetic) feature of micronuclei in the course of macronuclear differentiation. Acetylation of H3K9 or H3K14 was not observed in micronuclei (m) at any developmental stage (Figure 4A, B; Figure 5A, B; Figure 6A, B).

Macronuclear anlagen at early developmental stages (stages a_1_/early a_2_, compare Figure 1) were morphologically characterized by the decondensation of chromatin, accompanied by the appearance of H3K9ac (Figure 4A) and H3K14ac (Figure 4B). Western analyses confirmed that H3K9ac/K14ac was abundant ~24 hours post conjugation (stage a_2_) in macronuclear anlagen stages (Additional file 2A). It appeared that for both modifications signals were restricted to the interior of the early macronuclear anlage, while the condensed chromatin at the nuclear periphery remained unstained. This observation is consistent with earlier microscopic studies, which showed that only a portion of the chromosomes in the early macronuclear anlagen move to the center of the nucleus, are despiralized and develop into polytene chromosomes, while the residual chromosomes become pyknotic and are lost from the macronuclear anlage [13,16].

While H3S28p accumulated in the early macronuclear anlagen stages (a_1_) (Figure 4J), we could not detect H3K9me3 (Figure 4G), H3K27me1 (Figure 4H), or H3K27me3 (Figure 4I) in this stage (a_1_/early a_2_) by immunofluorescence. These observations highlight that the fusion of two heterochromatic micronuclei during conjugation leads to a zygote nucleus, in which all repressive micronuclear-specific histone modifications are removed and permissive histone modifications are introduced *de novo*.

However, repressive methylation hallmarks on histone H3 were shown to be associated with micronucleus-specific sequences during macronuclear differentiation in ciliates such as *Tetrahymena *[21,22,31] or *Stylonychia *[18]. These modifications are a necessary prerequisite for correct DNA processing. Whereas we did not find repressive methylation markers in early macronuclear anlagen (a_1_), our microscopic data show that after transition to the next developmental stage (early a_2_) sporadic foci-like signals of these modifications were observed in the anlage (for example, H3K9me3 in Figure 4G and H3K27me3 in Figure 4I). In addition, the *de novo *introduction of H3K36me3 was detected in macronuclear anlagen at stage a_2 _of polytenization (Figure 4E). Western analyses confirmed that this modification occurred in macronuclear anlagen (a_2_) ~24 hours post conjugation (Additional file 2A). Due to asynchronous macronuclear differentiation in mass cultures, performance of quantitative normalized Western blot analyses was not possible. We therefore determined the ratios of H3K9ac/K14ac and H3K27me3 to DNA in microscopic sections of macronuclear anlagen in various stages of development (Additional file 3). Although it is generally difficult to compare fluorescence intensities of different antibodies directly, these measurements were a valuable relative approximation to plot the time lapses of H3K9ac/K14ac or H3K27me3 signals, respectively, confirming that H3K9ac/K14ac accumulated early during macronuclear differentiation whereas H3K27me3 signals remained on a threshold level at that time.

#### Repressive histone modifications occur in macronuclear anlagen with high levels of polyteny and are lost in the course of DNA elimination

**Figure 7:**
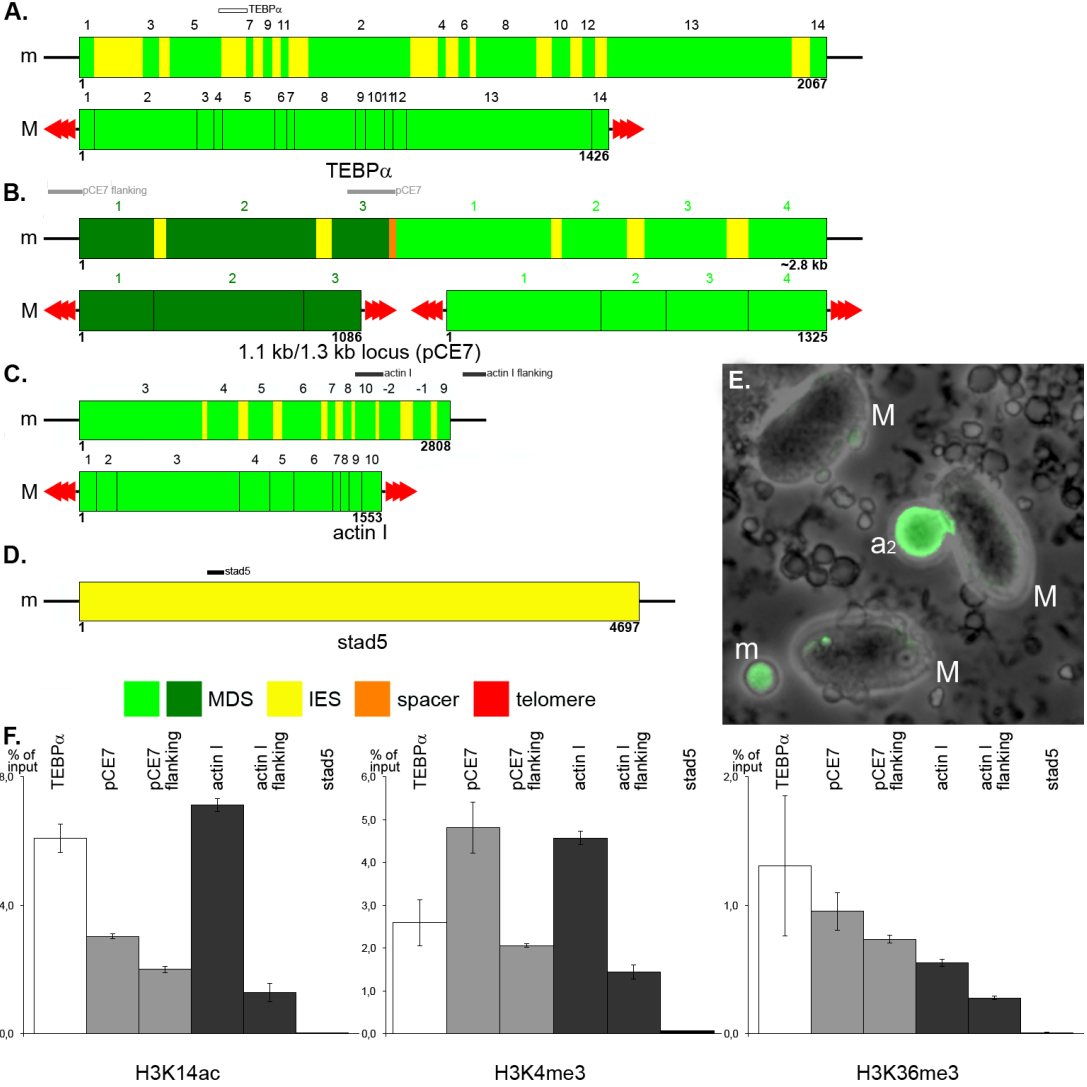
**ChIP analyses of permissive histone modifications in macronuclear anlagen**. **(A) **Diagram of the micronuclear and the macronuclear version of the *Stylonychia *TEBPα gene. MDSs occur in scrambled disorder (1-3-5-7-9-11-2-4-6-8-10-12-13-14) in the micronucleus. For real-time PCR analyses primers from MDS3 (Mi2) and IES3 (IES3) were used. **(B) **Diagram of the micronuclear and the macronuclear version of the *Stylonychia *1.1 kb and 1.3 kb locus. For real-time PCR analyses of the MDSs primers from the 1.1 kb (P94) and the 1.3 kb (P95) locus were selected. For real-time PCR analyses of the flanking sequence primers Sz1 and Sz2 were used. **(C) **Diagram of the micronuclear and the macronuclear version of the *Stylonychia *actin I gene. MDSs occur in scrambled disorder (3-4-5-6-7-8-10- [-2]- [-1]-9) in the micronucleus, furthermore MDSs 1 and 2 are inverted. For real-time PCR analyses of the MDS primers from MDS10 (Actinfor) and MDS-2 (Actinrev) were used. For real-time PCR analyses of the flanking sequence primers Actin5f and Actin5r were selected. **(D) **Diagram of the micronucleus specific sequence stad5. For real-time PCR analysis primers Stad5-3654 and Stad5-3520 were used. **(A-D) **Positions of PCR fragments are marked above the diagrams. **(E) **Fluorescence *in situ *hybridization on isolated nuclei using stad5 as a probe. Stad5 (green) could be detected in micronuclei (m) and macronuclear anlagen (stage a_2_), but not in macronuclei (M). **(F) **Quantitative real-time PCR analyses of ChIP experiments. Primer sequences are listed in Methods.

As described above, H3K9ac and H3K14ac are introduced *de novo *in the macronuclear anlage immediately after division of the zygote nucleus. Both modifications were abundant in later stages of macronuclear development, including advanced stages a_2_/a_3 _with a high degree of polytenization (Figure 5A, B), and stages (e) during DNA-elimination (Figure 6A, B) up to and including the mature macronucleus (Figure 2A, B). Western analysis confirmed that H3K9ac/K14ac was abundant ~48 hours post conjugation (transition stage a_3 _→ e) in advanced macronuclear anlagen stages (Additional file 2A). While H3K9ac and H3K14ac appeared to be evenly distributed over the entire interior of the macronuclear anlagen in stages a_1 _and a_2 _(Figure 4A, B), large chromatin domains were unstained in the advanced macronuclear anlagen stages (a_3_) (Figure 5A, B). This became even more obvious in single light optical sections (Figure 5K), where large chromatin domains exhibited no H3K9ac/K14ac signals. These domains corresponded to 'heterochromatic blocks' of polytene chromosomes described earlier [13]. According to the measurements of the H3K27me3:DNA ratio in microscopic sections it could be roughly estimated that at the late polytene chromosome stage up to 80% of the DNA pixels co-localized with H3K27me3 signals. We performed ChIP experiments using antibodies specific for H3K9ac and H3K14ac respectively. Subsequently DNA fragments from three sequence classes were analyzed by real-time quantitative PCR (Figure 7A–D) using three macronucleus-destined sequences encoding the TEBPα gene (GeneBank accession number: FJ159127, the 1.1 kb and 1.3 kb macronuclear nanochromosome pCE7; GeneBank accession number: X72958; [18] and the actin I gene GeneBank accession number: DQ108616); flanking sequences from the micronuclear 1.1 kb locus and the actin I gene; and the moderate repetitive sequence 'stad5' (Gene Bank accession number: EU687742), a micronuclear genomic sequence, which is specifically eliminated during macronuclear development (Figure 7D; Additional file 2B). Most analyses in the holotrichous ciliate *Tetrahymena *concentrated on the elimination of IES, which have a length up to several kilo base pairs. In contrast, IES size in stichotrichous ciliates like *Stylonychia *ranges between 1 to 100 bp [12]. Because of this property we did not pursue analyses of IES chromatin structure and elimination in the present study. In the germline TEBPα and actin I genes multiple MDSs, interrupted by micronucleus specific DNA, occur in a scrambled disorder [54] (Figure 7A). To exclude amplification from macronuclear contamination, DNA primers derived from an IES and a MDS (TEBPα), from the 3' end of the 1.1 kb and 5' end of the 1.3 kb locus (pCE7) and a scrambled part of the micronuclear actin I sequence were used for PCR analyses (Figure 7A to 7C). Our ChIP experiments revealed that H3K14ac was associated with all MDSs (Figure 7F). To a significantly lower extent we found these modifications associated with sequences flanking the 1.1 kb locus and the actin I gene. For the micronucleus-specific stad5 no association with H3K14ac (Figure 7F) was observed. Similar results were obtained using the H3K9ac-specific antibody for ChIP (data not shown).

During the first rounds of DNA amplification immunofluorescence signals of H3K4me1 (Figure 5C) and H3K4me3 (Figure 5D) were observed to drastically accumulate *de novo *in advanced stages (a_3_) of macronuclear development. H3K4me1 (Figure 6C) and H3K4me3 (Figure 6D) were also present during the DNA elimination stage (e) and were retained in the mature macronucleus (Figure 2C, D). The presence of both modifications in macronuclear anlagen ~48 hours post conjugation (transition stage a_3 _→ e) was also demonstrated by Western analyses (Additional file 2A). ChIP experiments using antibodies specific for H3K4me3 demonstrated that this modification was associated with the MDSs and to a lesser extent with their flanking sequences. The association with stad5 was negligible (Figure 7F).

H3K36me3 was detected by immunofluorescence microscopy (Figure 5E) and Western analysis (Additional file 2A) at advanced macronuclear anlagen (a_3_) with high level of polyteny. An accumulation of H3K36ac was also observed at this stage using *in situ *antibody staining (Figure 5F). However, in contrast to H3K4 methylation, H3K36me3 was no longer observed in the subsequent DNA elimination stage (e), indicating the loss of H3K36me3 during DNA elimination (Figure 6E). Similarly, H3K36ac (Figure 6F) exhibited maximum signal intensity at the transition from highly polytene anlagen to the elimination stages (a_3_-e), and during the second round of DNA amplification we could not detect this modification. ChIP experiments using antibodies directed against H3K36me3 demonstrated that this modification was associated with MDSs and to lesser extent with sequences in the flanking regions of the MDSs. An association with stad5 was not observed (Figure 7F).

H3S28p persisted during polytenization in macronuclear anlagen (a_3_) (Figure 5J) and disappeared during DNA elimination (e) (Figure 6J), and was not retained in mature macronuclei (M) (Figure 2J). ChIP experiments using antibodies specific for H3S28p demonstrated that this modification was associated with the MDS of actin I. To a lesser extent H3S28p was observed to associate with actin I flanking sequence, whereas the association with stad5 was marginal (Additional file 4).

Earlier studies demonstrated that sequences targeted for elimination during macronuclear development are associated with histone modifications typically found within silenced chromatin, such as H3K9me3 and H3K27me3 in *Tetrahymena *[21,22,31] or H3K9me3 in *Stylonychia *[18]. Our immunofluorescence analyses showed that *de novo *tri-methylation at H3K9 (Figure 5G) and mono-methylation at H3K27 (Figure 5H) take place at advanced stages of polytenization in macronuclear anlagen (a_2_, early stage a_3_). Both modifications were still detected with gradually decreasing signal intensity during the DNA elimination stage (e) (Figure 6G; Figure 6H), but were not observed during the second rounds of DNA amplification.

H3K27me3 exhibited the most prominent signals of repressive histone modifications examined in macronuclear anlagen (a_3_) late during polytene chromosome formation (Figure 5I). Single light optical sections (Figure 5L) showed that large chromatin domains exhibited H3K27me3 signals exactly where H3K9ac/K14ac signals were obviously omitted (compare Figure 5K). In spread polytene chromosome preparations it became obvious that H3K27me3 was enriched in chromosomal bands as well as in 'heterochromatic blocks' (Figure 5M). H3K27me3 was observed to occur with decreasing signal intensity during stages (e) of DNA elimination (Figure 6I; Additional file 3).

**Figure 8:**
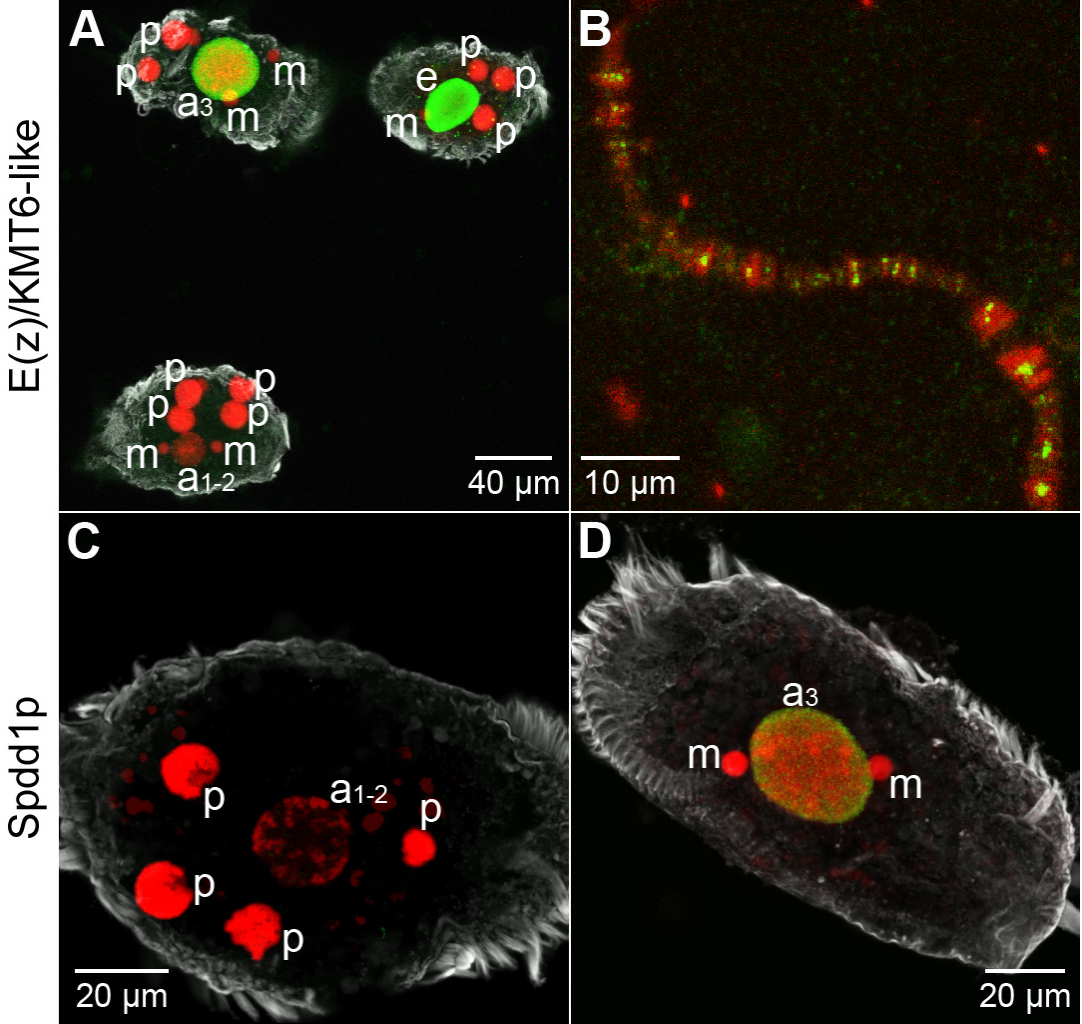
**Chromatin-modifying proteins during macronuclear differentiation**. *In situ *antibody staining of a putative E(z)/KMT6-like protein **(A, B) **and the HP1-homolog Spdd1p **(C, D) **(green). To-Pro-3 was used for DNA counterstaining (red). The cellular shape was visualized using an α-tubulin-antibody (grey). Confocal image stack projections. **(A) **In macronuclear anlagen the E(z)/KMT6-like protein is not detected at stage a_1–2_, but accumulates subsequently (a_3_) and is present at stage e during DNA elimination. **(B) **The E(z)/KMT6-like protein co-localizes with chromosomal bands in spread polytene chromosome preparations. **(C) **Spdd1p is not detected in early macronuclear anlagen (a_1–2_). **(D) **Spdd1p accumulates in advanced stages (a_3_) during macronuclear development.

Since it is well established that the selective introduction of H3K9me3 and H3K27me3 is a necessary prerequisite for the programmed elimination of DNA during macronuclear differentiation, we decided to analyze the spatiotemporal occurrence of two chromatin modifying proteins, a putative histone methyltransferase (HMT) and the HP1 homolog Spdd1p, which have been identified previously in ciliates [21,55,56]. Initially, we decided to analyze the nuclear localization of a putative E(z)-like HMT (new nomenclature: K-methyltransferase 6/KMT6 [57]), since we assumed that homologs of this protein could be involved in the establishment of H3K9me3 and H3K27me3 in *Stylonychia*. The SET-domain-containing PcG protein E(z)/KMT6 or homologous proteins were identified to be core components of all polycomb repressive complexes 2 (PRC2) isolated so far. PRC2 complexes have HMT activity and are capable of transferring methyl groups at H3K27 and/or H3K9 [2,4]. An E(z)/KMT6 homolog has recently been identified in the ciliate *Tetrahymena *[21]. We applied an antibody targeted to EZH2 (Abcam; ab3748) for immunofluorescence microscopic analysis (Figure 8A). Although we could not detect any signals at early macronuclear anlagen stages (a_1–2_), we observed an accumulation of signals at advanced macronuclear stages (a_3_) during polytene chromosome formation. These signals persisted up to and including the DNA elimination stage (e), but were not retained during the second rounds of DNA amplification and in nuclei of vegetative *Stylonychia *(data not shown). Further immunofluorescence analyses on spread polytene chromosomes revealed that the protein detected with an anti-EZH2 antibody had a similar distribution to H3K27me3 (Figure 8B). However, the specificity of the antibody used still has to be shown in *Stylonychia*.

Heterochromatin formation in mammalian and *Drosophila *cell nuclei involves the recruitment of HP1, which specifically binds to H3K9me3 via its chromodomain [58-60]. The chromodomain-containing proteins Pdd1p in *Tetrahymena *[22,55] and Spdd1p in *Stylonychia *[56] are homologues of HP1 and both were shown to be involved in programmed DNA elimination. We therefore reinvestigated the occurrence of Spdd1p during *Stylonychia *macronuclear development. It has been shown that during macronuclear differentiation Spdd1p is present in the macronuclear anlagen as well as in the degenerating parental macronuclei [56]. Our immunofluorescence analyses confirmed that Spdd1p occurred in macronuclear anlagen with a similar pattern to H3K9me3 or H3K27me3 (Figure 8). Spdd1p was detected neither in micronuclei nor in macronuclei of vegetative *Stylonychia *(data not shown). In early macronuclear anlagen (a_1–2_) no signals were observed (Figure 8C) before Spdd1p accumulated in macronuclear anlagen (a_3_) at later stages of polytenization (Figure 8D) and was present during the DNA elimination stage (e) (data not shown).

In summary, our data show that repressive histone modifications are introduced *de novo *at later stages during macronuclear differentiation, and they are found in the banded regions and heterochromatic blocks of the polytene chromosomes. While these modifications are lost during the DNA elimination stage, 'open' chromatin markers introduced at very early stages persist throughout macronuclear differentiation up to the vegetative macronucleus.

#### The PIWI-family protein Piwi/mdp1 exhibits alternate accumulation in different nuclear types in the course of macronuclear development in *Stylonychia*

**Figure 9:**
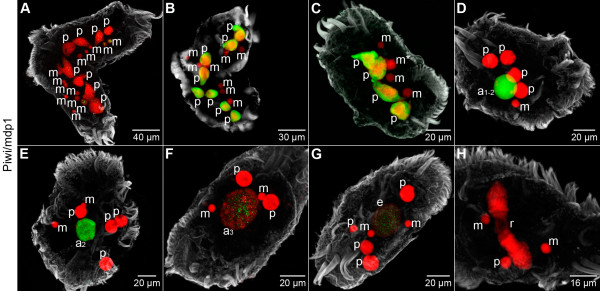
**A PIWI-family is involved in a trans-nuclear cross-talk**. Immunofluorescence of Piwi/mdp1 (green) and DNA counterstaining (red). The cellular shape was visualized using an α-tubulin-antibody (grey). Confocal image stack projections. **(A) **Early during conjugation Piwi/mdp1 accumulates in a subset of micronuclei (m), and some scattered foci-like signals are observed in the fragmented parental macronuclei (p). During conjugation **(B)**, and early after separation of conjugating cells **(C)**, Piwi/mdp1 is highly enriched in the fragmented parental macronuclei (p) but is no longer detected in micronuclei (m). **(D-H) **At subsequent developmental stages Piwi/mdp1 signals totally vanish from the fragmented parental macronuclei (p). **(D) **Instead Piwi/mdp1 relocalizes in the macronuclear anlage and exhibits maximum signal intensity at an early stage (a_1–2_) during the first DNA amplification stage. **(E, F) **As polytenization proceeds (a_2 _in **(E) **and a_3 _in **(F)**), Piwi/mdp1 signals becomes organized as discrete foci. **(G) **Scattered Piwi/mdp1 signals are still detected at stages (e) during DNA elimination. **(H) **At stages (r) during the second rounds of DNA amplification no more Piwi/mdp1 signals are observed.

Direct evidence for the involvement of an RNAi-related machinery in the control of DNA elimination by heterochromatin formation has been recently provided [7-9,18,19], and it has been shown that the Piwi-family protein Twi1p involved in the trafficking of small RNA molecules in *Tetrahymena *is an important component of that pathway [7,9]. In *Stylonychia *the Piwi-family macronuclear development protein 1 (mdp1, predicted size ~89 kDa) is differentially expressed during macronuclear development [25,26]. Besides the PIWI-domain, which is thought to be involved in protein-protein interactions, *Stylonychia *Piwi/mdp1 possesses a putative RNA-binding PAZ domain, suggesting that this protein is involved in the processing of small RNA molecules, which have been shown to be complementary to micronucleus specific sequences [18]. We therefore analyzed in detail the spatiotemporal occurrence of Piwi/mdp1 during macronuclear development by immunofluorescence microscopy (Figure 9) as well as Western analyses (Additional file 2A). Using RNAi directed against various regions of the Piwi/mdp1 gene, the intensity of the putative Piwi/mdp1 band was greatly reduced (Additional file 2A), and as reported earlier [27] an arrest of macronuclear development at an early stage was observed. Immunofluorescence analyses showed that Piwi/mdp1 accumulated in a sub-fraction of micronuclei (m) early during conjugation (Figure 9A), whereas the *Tetrahymena *homologous protein Twi1p was observed in the cytoplasm at comparable stages [7]. Coevally we observed the accumulation of scattered foci-like signals in the fragmented parental macronuclei (p). Later during conjugation (Figure 9B) a high signal intensity of Piwi/mdp1 was found in the fragmented parental macronuclei (p), while it was no longer present in micronuclei (m). Piwi/mdp1 was still present in the fragmented parental macronuclei (p) immediately after separation of conjugating cells (Figure 9C). Subsequently Piwi/mdp1 signal density drastically decreased in these parental macronuclear fragments (p) but relocalized to the macronuclear anlagen (a_1–2_) early during polytenization (Figure 9D). We detected a maximum Piwi/mdp1 signal intensity in macronuclear anlagen at this stage, and the staining appeared to be homogenously distributed. Piwi/mdp1 signals in macronuclear anlagen (a_2_, a_3_, e) were observed to become organized as discrete foci, and the signal density continuously decreased in the course of further polytenization (Figure 9E, F) and during DNA elimination (Figure 9G). Subsequently we observed that Piwi/mdp1 was completely lost from developing macronuclei (r) during the second round of DNA amplification (Figure 9H).

## Discussion

The most extreme DNA elimination and reorganization processes observed so far in nature take place during macronuclear differentiation in stichotrichous ciliates, such as *Oxytricha *or *Stylonychia*. The resulting somatic macronucleus contains only a small yet highly amplified fraction of the generative micronuclear genome. We show here that very early during macronuclear differentiation DNA sequences to be retained become and stay associated with histone markers typically associated with 'open' chromatin. Repressive chromatin hallmarks that have been shown to specify sequences for elimination in *Stylonychia *become introduced only at later stages [18].

DNA elimination can be regarded as an extreme form of DNA sequence silencing. Therefore both processes, developmental programmed DNA elimination in ciliates and the specific repression of genes during early development in metazoan, might require similar mechanisms. The nuclear dualism found in ciliates, where a silent germline micronucleus and a highly active somatic macronucleus occur within a single cell, and the morphologically well-characterized macronuclear differentiation process in the ciliate *Stylonychia *provide a unique biological model system for the analysis of chromatin plasticity during a differentiation process in eukaryotes.

### Each nuclear type possesses its specific histone modification signature

In this study we analyzed the histone modification patterns of the two specialized vegetative nuclei, the germline micronucleus and the somatic macronucleus, and subsequently focused on the characterization of the spatial and temporal dynamics of histone modifications during macronuclear development and their biological relevance for nuclear differentiation. We also examined proteins regulating chromatin structure and a protein involved in the RNAi-mediated heterochromatin formation. We found that each type of nucleus is characterized by a specific histone signature, and that the chromatin status of the developing macronucleus is spatially and temporally highly regulated.

Many of the histone modifications known to occur in the cell have been related to transcriptional activity or repression, although exceptions for almost all modifications have been reported [2]. In line with previous studies of metazoan cell nuclei, the DNA of the transcriptionally inert and heterochromatic micronucleus is associated with a histone modification typical for repressed chromatin, H3K27me3 (Figure 2I), but no H3K9me3 (Figure 2G) or H3K27me1 (Figure 2H), classical hallmarks for constitutive heterochromatin could be found in this nucleus. In contrast to the heterochromatic micronucleus, the transcriptionally active macronucleus exhibits only histone modifications typical for transcriptionally competent or 'open' chromatin (Figure 2A to 2D).

### A permissive chromatin state characterizes early macronuclear differentiation, and at advanced developmental stages repressive chromatin markers are introduced selectively

Our most striking observation was made in very early stages of macronuclear differentiation. At these stages repressive histone markers characteristic for the germline micronucleus, such as H3K27me3 (Figure 4I), are removed and 'open' chromatin hallmarks such as acetylated histone H3 (Figure 4A, B) are introduced *de novo*. This is reminiscent of the early embryonic cell nuclei of metazoa, in which chromatin apparently adopts an open, hyperdynamic state [61]. To our knowledge detailed information about the histone language in early embryonic cells is still rather limited. As differentiation proceeds, repressive hallmarks have been reported to accumulate in a number of biological systems [62-64]. For example, it has been observed in mice that during the first zygotic cell divisions genomic DNA becomes progressively hypomethylated. It has also been described that repressive epigenetic markers such as DNA methylation as well as H3K9me and H3K27me are *de novo *introduced during and after the 8 to 16 cell stage, when the outer morula cells flatten and can be distinguished from the inner cell mass as a first morphological sign of cell differentiation [65,66]. Concomitantly heterochromatin spatially accumulates in the course of differentiation [64,67].

Similar to these observations made in metazoa, we now show that during successive rounds of replication in the developing macronucleus repressive histone markers become introduced *de novo *in a spatially and temporally highly regulated manner. Earlier ChIP analyses performed in our laboratory demonstrated that in *Stylonychia *exclusively micronucleus-specific sequences, which become eliminated during macronuclear development, are associated with H3K9me3 [18], as has also been shown in the holotrichous ciliate *Tetrahymena *[22,31]. Our current analyses show that MDSs in macronuclear anlagen remain associated with H3K9ac/K14ac introduced after zygote nucleus formation (Figure 7F). This is consistent with previous electron microscopic observations on chromatin of spread macronuclear anlagen: in the course of polytenization chromatin becomes organized in heterochromatic loops bound to a chromatin axis in its open chromatin status [16,17].

An E(z)/KMT6-like HMT, a putative enzyme required to transfer methyl groups at H3K9 and H3K27, was observed to accumulate during the first round of DNA amplification in the course of macronuclear development (late stage a_2_/a_3_) (Figure 8A). Repressive histone modifications introduced during macronuclear differentiation include H3K9me3 (Figure 5G), H3K27me1 (Figure 5H), and H3K27me3 (Figure 5I). Simultaneous with the appearance of these repressive histone modifications, the heterochromatin specific protein Spdd1p, whose *Tetrahymena *homolog Pdd1p binds to H3K9me3 and H3K27me3 [21,22], is detectable in the macronuclear anlagen (Figure 8C, D). In macronuclear anlagen containing polytene chromosomes it became obvious that repressive histone modifications such as H3K27me3 (Figure 5M) as well as the E(z)/KMT6-like HMT (Figure 8B) are exclusively localized in the banded regions and heterochromatic blocks of the polytene chromosomes. All repressive histone modifications examined are lost in the course of DNA elimination (Figure 7G to 7I).

In contrast to these repressive histone modifications, 'open' chromatin hallmarks H3K9ac/K14ac occur very early in macronuclear anlagen, persist throughout macronuclear development and are still found in the mature macronucleus (Figure 2A, B; Figure 4A, B; Figure 5A, B; Figure 6A, B). In macronuclear anlagen antibodies directed against these modifications neither stained the banded regions nor the heterochromatic blocks of polytene chromosomes, and therefore these modifications are probably localized in the interbands of the polytene chromosomes, but cannot be detected on the level of resolution used here. It has been shown earlier that inhibition of HDAC activity using trichostatin A leads to an arrest in macronuclear differentiation and to severely disturbed repressive histone methylation [18,28]. These observations show that histone deacetylation prior to *de novo *methylation of H3 is an essential step for specific processing of DNA sequences during this nuclear differentiation process. The requirement of deacetylase activity for macronuclear differentiation confirms that global histone hyperacetylation occurs at very early macronuclear developmental stages.

Unlike acetylation, H3K4me1 and H3K4me3 are introduced *de novo *at an advanced stage of polytenization during macronuclear development. While H3K4me3 was preferentially located at compartment borders separating condensed and apparently chromatin-free regions in the macronucleus (Figure 2L), H3K4me1 foci were evenly distributed within macronuclear chromatin (Figure 2K). Therefore H3K4me1 is possibly linked to euchromatic gene silencing. Our ChIP analyses demonstrate that H3K4me3 in advanced macronuclear anlagen (Figure 7F) is specifically targeted to sequences, which are retained in the macronucleus. However, a targeting mechanism remains unknown.

The function of H3K36me3 remains enigmatic. This modification was present in vegetative micronuclei (Figure 2E), but lost during conjugation (Figure 3E). Subsequently H3K36me3 (Figure 4E, Figure 5E) was introduced *de novo *in macronuclear anlagen and lost in the course of DNA elimination (Figure 6E). ChIP analyses revealed that H3K36me3 is associated with MDSs in macronuclear anlagen (Figure 7F). It may well be possible that H3K36me3 could antagonize erratic initiation of transcription in micronuclei and macronuclear anlagen, similar to a proposed function described earlier in *S. cerevisiae*. Here, H3K36me3 is assumed to prevent the internal initiation of transcription towards the 3' end of genes by recruitment of a histone deacetylase [2,42]. The loss of H3K36me3 during conjugation in *Stylonychia *may be a necessary prerequisite for the *de novo *acetylation of histones in the early macronuclear anlage. Remarkably, we could not observe H3K36me3 at the gene-sized macronuclear nanochromosomes, suggesting that this 'gene marker' is dispensable with this type of genome organization. The loss of H3K36me3 during macronuclear development marks the sole exception that a gene-associated marker is not retained in the mature macronucleus. Noteworthy, the discovery that H3K36me3 is present in *Stylonychia *is novel in ciliates.

H3 phosphorylated at Ser-28 occurs in vegetative macronuclei (Figure 2J), suggesting a role for this modification in transcriptional activity, as has been reported very recently for nuclei of immature chicken erythrocytes [53]. Furthermore, H3S28p is abundant during macronuclear developmental stages (stages a_1–3_, Figure 4J; Figure 5J; stage e, Figure 6J), and our ChIP analyses suggest that this modification is associated with macronucleus destined actin I sequences in macronuclear anlagen (Additional file 4). It has been demonstrated that phosphorylation of Ser-10 prevents or disrupts binding of HP1 to adjacent H3K9me3 [49,50]. This could also apply for H3S28p, which may be introduced in macronuclear anlagen to prevent heterochromatin spreading by undesirable binding of Spdd1p to H3K27me3 at sequences which need to be retained in the vegetative macronucleus. In fact, it has been observed in *Tetrahymena *that the mutation of H3S28 (H3S28E) disrupts the proper methylation of H3K9 and H3K27 during macronuclear development [21]. Our present data on H3S28p thus contribute to the emerging view that phosphorylation of serine residues adjacent to methylated lysines, such as H3K9me3, possibly H3K27me3 and also H1K26me3 [51], can act to modulate binding of effector proteins such as HP1 or homologous proteins. Thus serine phosphorylation can directly affect the establishment of chromatin states. Notably, H3S28p occurrence was demonstrated for the first time in ciliates, and the importance of this histone modification for a nuclear differentiation process has not been described before.

Taking all these observations together we demonstrate that sequences which become eliminated during macronuclear differentiation are targeted to heterochromatin formation by the introduction of repressive histone modifications. In contrast, sequences which are destined to be retained in the macronucleus stay associated with permissive chromatin modifications, which are introduced *de novo *during early stages of macronuclear differentiation, immediately after fusion of the two heterochromatic micronuclei.

### The PIWI family protein Piwi/mdp1 is involved in a transnuclear cross-talk and possibly acts as an interface between an RNAi pathway and heterochromatin formation of micronucleus-specific sequences

In recent years strong evidence has emerged for the existence of an RNAi-dependent mechanism regulating the specific excision and elimination of micronucleus-specific DNA sequences. Although the exact mechanisms are not yet understood and differences between different ciliate species may occur, it seems that small RNA molecules result from bi-directional transcription of micronuclear DNA early during conjugation. These small RNA molecules are transported to the fragmented parental macronucleus where selection of small RNA molecules takes place resulting in an accumulation of small RNA molecules homologous to micronucleus-specific DNA sequences. These so-called scanRNAs invade the macronuclear anlage and specify the sequences to be removed [7-9,18-20]. It has been assumed that proteins of the PIWI family are involved in RNA trafficking and could possibly act as a recruitment platform for chromatin-modifying enzymes, but evidence for a direct interaction is moderate to date. However, it has been reported in *Drosophila *that Piwi specifically interacts with HP1a, and that this interaction appears to be RNA-dependent [68].

During conjugation of *Stylonychia *a PIWI family protein (Piwi/mdp1) first accumulates in a subset of micronuclei and subsequently is exclusively found in the fragmented parental macronucleus. Later this protein completely disappears from these nuclear fragments, and Piwi/mdp1 then relocates in the macronuclear anlage at an early stage of development (stage a_1–2_). During later developmental stages continuously decreasing signal intensity is observed, and after DNA elimination Piwi/mdp1 is no longer detectable (Figure 9). Our microscopic analyses demonstrate that the appearance of Piwi/mdp1 in the macronuclear anlagen strikingly coincides with the introduction of heterochromatin hallmarks. Our observations support the hypothesis that PIWI family proteins are involved in the trafficking of small RNA molecules between the different types of nuclei as it has been reported in *Tetrahymena *[7]. As a consequence Piwi proteins might act as an interface between the cellular RNAi-dependent machinery and chromatin-modifying proteins.

## Conclusion

The observations described in this study extend earlier reports about the epigenetic control of macronuclear differentiation and allow a temporal and spatial characterization of epigenetic events occurring during this differentiation process. They can therefore serve as a guideline for further functional analyses on the molecular events occurring during a nuclear differentiation process, not only in ciliated protozoa but also in metazoan cells.

As in *Tetrahymena*, a bidirectional transcription of the micronuclear genome takes place in *Stylonychia *and we now show that Piwi/mdp1 relocates from the micronucleus to the old macronucleus and finally to the macronuclear anlagen, which supports the model that Piwi-like proteins are involved in the trafficking of small RNAs. Immediately after the fusion of the two heterochromatic micronuclei and mitotic division of the resulting zygote nucleus, a global chromatin decondensation in the macronuclear anlage takes place by the removal of all repressive micronucleus-specific histone modifications and *de novo *acetylation. As macronuclear development progresses, Piwi-bound small RNA molecules complementary to micronuclear-specific sequences invade the macronuclear anlage and specify sequences which are targeted to heterochromatin formation by *de novo *lysine methylation of H3K9 and H3K27 and subsequent binding of Spdd1p. These modifications are found exclusively in the banded regions and heterochromatic blocks of the polytene chromosomes. In contrast, sequences to be retained in the macronucleus stay associated with chromatin hallmarks typical for 'open chromatin'. This implies that sequences to be eliminated become organized in the banded regions of the chromosome, while the macronuclear genes are contained in the interbands. In the course of DNA elimination all repressive markers are lost, probably by selective degradation of the banded regions of the polytene chromosomes, while the 'active' markers stay associated with MDSs and are still found in the mature differentiated macronucleus.

## Methods

### Growth of *Stylonychia lemnae*

Growth of *Stylonychia lemnae *and isolation of micronuclei, macronuclei and macronuclear anlagen was performed as described elsewhere [13]. For mating, cells of two different mating types were mixed and allowed to conjugate.

### SDS Page and Western Analyses

For extraction of nuclear proteins, the various nuclei types were isolated (approximately 5 × 10^4^), collected by centrifugation, resuspended in loading buffer [69], heated for 10 min at 100°C, and separated on 15% sodium dodecyl sulfate (SDS) polyacrylamide gels. Proteins were then transferred onto a nylon membrane and probed with specific antibodies. Detection was performed using the digoxigenin system (Roche).

### Immunofluorescence

Cells containing nuclei at various developmental stages were fixed in 2% paraformaldehyde for 20 min at room temperature, washed twice with phosphate buffered saline (PBS), and immobilized onto poly-L-lysine-coated coverslips. Subsequently cells were permeabilized with 0.5%TritonX100 in PBS for 20 min, followed by incubation with 0.1N HCl for 5 min at room temperature. For some experiments polytene chromosomes were spread onto microscopic slides: whole cells were gently separated ~48 hours post conjugation using a Heraeus Megafuge at 1000 rpm, 4°C, 7 min. Cells were resuspended in methanol:acetic acid (3:1) and incubated overnight. Polytene chromosomes were spread on adhesion microscopic slides (Menzel SuperFrost^® ^Plus) by iterative dropping from 20 to 30 cm height. The drop was subsequently dispersed using a focused labial air flow generated by a forceful exhalation.

Blocking was done in 4%BSA, 0.1%TritonX100, PBS for 20 min at room temperature. Antibodies were diluted in blocking solution. Between application of primary and secondary antibodies washing steps were performed in PBS for 20 min. Incubations with all primary antibodies were performed overnight at 4°C; subsequent incubations with all secondary antibodies were performed 1.5 h at 37°C. A list of primary antibodies targeted to histone modifications, chromatin-modifying enzymes, a Piwi-like protein and 5-FU used in our experiments is found in Table 2. Goat anti-mouse or goat anti-rabbit polyclonal antibodies conjugated with either Alexa Fluor 488 or Alexa Fluor 555 (Molecular Probes) or goat anti-rat-Cy3 polyclonal antibodies (Amersham) were used for secondary detection. The cell shape was visualized using antibodies targeted to α-tubulin (either Sigma T9026 DM1α/mouse monoclonal or Abcam ab4074/rabbit polyclonal, depending on the host species of other primary antibodies used). Nuclei were counterstained with 0.1 μg/ml 4',6-diamidino-2-phenyl-indole (DAPI) (Sigma) and 1 μM To-Pro-3 (Molecular Probes) in PBS. Preparations were mounted in Vectashield antifade medium (Vector Laboratories) and sealed with nail polish.

**Table 2:** Antibodies used in this study.

	*Manufacturer*	*Product code/Reference*
***Histone acetylation***		
*H3K9ac*	Abcam	#12179
*H3K14ac*	Upstate	#07–353
*H3K9ac/K14ac*	Santa Cruz	#sc-8655-R
*H3K36ac*	Upstate	#07–540

***Histone methylation***		
*H3K4me1*	Abcam	#ab8895
*H3K4me3*	Abcam	#ab8580
*H3K9me3*	Antoine Peters, FMI Basel, CH	Peters, AH et al., Mol Cell (2003)
*H3K27me1*	Antoine Peters, FMI Basel, CH	Peters, AH et al., Mol Cell (2003)
*H3K27me3*	Antoine Peters, FMI Basel, CH	Peters, AH et al., Mol Cell (2003)
*H3K36me3*	Abcam	#ab9050

***Histone phosphorylation***		
*H3S28p*	Abcam	#ab5169

***Chromatin modifying enzymes***		
*E(z)/KMT6-like HMT*	Abcam	#ab3748
*Spdd1p*	David Allis, Rockefeller University, USA	Madireddi, MT et al., Cell (1996)

***RNAi-related machinery***		
*Piwi/mdp1*	Abcam	#ab12337

### Peptide competition assay

To select antibodies for this study and to ensure their specificity we performed immunofluorescence-based peptide competition assays as described elsewhere [70].

### Confocal laser scanning microscopy

Nuclei were analyzed by CLSM. Acquisition of serial sections was done with a Leica TCP SP confocal laser scanning microscope (Leica Microsystems, Mannheim) equipped with an oil immersion objective lens (PL Fluortar 25/0.75, or in some cases HCX PL APO 63/1.32). Fluorochromes were visualized with an argon laser with excitation wavelengths of 488 nm for AlexaFluor488™ and 514 nm for AlexaFluor555™, and with a helium-neon laser with excitation wavelength of 633 nm for To-Pro-3. Fluorochrome images were scanned sequentially generating 8-bit grayscale images. Image resolution was 512 × 512 pixels with a pixel size ranging from 195 to 49 nm depending on the selected zoom factor. The axial distance between light optical serial sections was 500 nm. To obtain an improved signal-to-noise ratio each section image was averaged from four successive scans. The 8-bit grayscale single channel images were overlaid to an RGB image assigning a false color to each channel and then assembled into tables using open source software ImageJ (Rasband, W.S., ImageJ, National Institutes of Health, Bethesda, Maryland, USA, , 1997–2004.) and Adobe Photoshop CS3 software.

### Chromatin immunoprecipitation

To all buffers PMSF or protease inhibitor cocktail was added to avoid protein degradation. In some cases 5 mM sodium butyrate was added to avoid histone deacetylation. Cells were cross-linked for 10 min at room temperature using 1% formaldehyde. To stop the reaction 0.125 M glycine was added and incubated for 5 min. Cells were lysed in cell lysis buffer (0,1% Triton X-100, 200 mg/L spermidine, 500 mM sucrose) and macronuclear anlagen were collected on a 20 μm gauze. Nuclei were washed twice with cold PBS for 5 min and resuspended in nuclei lysis buffer (1% SDS, 10 mM EDTA, 50 mM Tris/HCl, pH8.0, protease inhibitor). Chromatin was sheared on ice to a length of approximately 500 to 1000 bp using a Branson 250 sonifier (settings: output 3, duty cycle 30%, 12 bursts for 10 sec each). The lysate was cleared by centrifugation (14,000 ×g, 5 min, 4°C). For each ChIP 25 μg DNA was diluted in 300 μl nuclei lysis buffer, filled up with 5 ml dilution buffer (1% Triton X-100, 150 mM NaCl, 2 mM EDTA, 20 mM Tris/HCl, pH8.0, protease inhibitor), and pre-cleared with Sepharose A beads for 2 h at 4°C. Beads were pelleted by centrifugation (150 ×g, 5 min, 4°C), and the supernatant was transferred to a new reaction tube. At this step one aliquot of pre-cleared chromatin was kept as 'input DNA' for future real-time PCR analysis. Sepharose A beads were blocked with BSA and sheared salmon sperm DNA for 2 h at 4°C in dilution buffer, and centrifuged (150 ×g, 5 min, 4°C). Immunoprecipitation was performed overnight using 3 μg of each specific antibody and blocked Sepharose A beads at 4°C at gentle rotation. Antibodies used for ChIP are listed in Table 2. Immunocomplexes were washed at gentle rotation at 4°C for 10 min as follows: twice with TSE I buffer (0.1% SDS, 1% Triton X-100, 2 mM EDTA, 500 mM NaCl, 20 mM Tris/HCl, pH8.1), twice with buffer III (0.25 M LiCl, 1% Nonidet P-40, 1% deoxycholate, 1 mM EDTA, 10 mM Tris/HCl, pH8.1) and once with TE buffer (2 mM EDTA, 10 mM Tris/HCl, pH8.0). To elute, immunocomplexes were incubated with elution buffer (1% SDS, 10 mM EDTA, 50 mM Tris/HCl, pH8.1) for 30 min at 65°C on a shaker. Eluted immunocomplexes were treated with proteinase K and incubated at 65°C for 6 h to reverse the crosslink. DNA was purified using phenol-chloroform extraction and ethanol precipitation.

### Quantitative real-time PCR

Quantitative real-time PCR (qRT-PCR) analyses were performed using a Light Cycler instrument (Roche Diagnostics). For PCR reactions a ready-to-use 'hot start' reaction mix (FastStart DNA MasterPLUS SYBR Green I; Roche Diagnostics) containing Taq DNA polymerase and SYBR Green I fluorescent dye for real-time detection of double-stranded DNA was used as recommended by the manufacturer. To generate standard curves for amplicons, input DNA of known concentration was serially diluted and analyzed in duplicates by qRT-PCR. Using individual standard curves for each amplicon, the relative amounts of specifically immunoprecipitated DNA analyzed by qRT-PCR in duplicates could be estimated as 'percent of input'.

The following primers were used for qRT-PCR analysis, (see Table 3).

**Table 3:** 

**Primer**	**Sequence**
TEBPα Mi2	5'-CTAAGAGATTTGAAGATCTTCCAATC-3'
TEBPα IES3	5'-CCATTCGTTTGTCATTTTGTTACTTATAATTAAATTATATC-3'
1.1 kb 1.3 kb P94	5'-CGCAACTTAATCTCAGAAAGCATA-3'
1.1 kb 1.3 kb P95	5'-GCAATTTTCCTGCCTTGTGT-3'
1.1 kb 1.3 kb Sz1	5'-TGCTCGAAAGACGAGATGTTC-3'
1.1 kb 1.3 kb Sz2	5'-GATTCATTATAAATTGGCTTTCCA-3'
Actinfor	5'-GCCAAGGACAGGTTGAATGA-3'
Actinrev	5'-TCGTCAAGGCTGGTTTAACA-3'
Actin5f	5'-AAGCTTCCTGGCTAATATTTTTGT-3'
Actin5r	5'-AGCAGATTAAAATTTTTCAAGTTA-3'
Stad5-3654	5'-CATCAAGGACCGCTATTCCTAC-3'
Stad5-3520	5'-AAAACATTCACCCCCAAAGC-3'

## Competing interests

The authors declare that they have no competing interests.

## Authors' contributions

JP carried out the microscopic analyses, performed the peptide competition assays and Western analyses of Piwi/mdp1, participated in the design and coordination of the study and in drafting the manuscript. KH carried out the ChIP experiments and Western analyses of modified histones and helped to draft the manuscript. MC and TC took part in the coordination of the study and helped to draft the manuscript. HJL participated in the design and coordination of the study and drafted the manuscript. All authors read and approved the final manuscript.

## Supplementary Material

Additional file 1 **Figure S1. Peptide competition assays**. Immunofluorescence microscopic analyses show the specificity of the antibodies used in this study. Grey-scale images were acquired using a Leica DM RB transmission light microscope equipped with a Leica DC300F CCD camera; subsequently false colors were assigned to each channel. Green: post-translational histone modifications. DAPI was used for DNA counterstaining (red). **(A) **H3K9ac antibody (ab); **(B) **H3K9ac ab competed with the immunizing peptide; **(C) **H3K14ac ab; **(D) **H3K14ac ab competed with the immunizing peptide; **(E) **H3K4me1 ab competed with a peptide trimethylated at K4; **(F) **H3K4me1 ab competed with the immunizing peptide; **(G) **H3K4me3 ab competed with a peptide monomethylated at K4; **(H) **H3K4me3 ab competed with the immunizing peptide; **(I) **H3K36me3 ab; **(J) **H3K36me3 ab competed with the immunizing peptide; **(K) **H3K36ac ab; **(L) **H3K36ac ab competed with the immunizing peptide; **(M) **H3K9me3 ab competed with a peptide trimethylated at K27; **(N) **H3K9me3 ab competed with the immunizing peptide; **(O) **H3K27me1 ab competed with a peptide trimethylated at K27; **(P) **H3K27me1 ab competed with the immunizing peptide; **(Q) **H3K27me3 ab competed with a peptide trimethylated at K9; **(R) **H3K27me3 ab competed with the immunizing peptide; **(S) **H3S28p ab; **(T) **H3S28p ab competed with the immunizing peptide. Click here for file

Additional file 2 **Figure S2**. **(A) **Western analyses confirm the occurrence of post-translational histone H3 modifications in micronuclei (m) and macronuclei (M) as well as in anlagen 24 h and 48 h post conjugation. Piwi/mdp1 (Western blot, left) is not detected in vegetative cell shortly before conjugation (0 h). A < 100 kDa protein is detected during macronuclear development (12 h and 24 h post conjugation) using an antibody targeted to a Piwi family protein. Western blot, right: The band detected with the Piwi antibody (lane wt) becomes weaker or disappears completely upon RNAi treatment (lane RNAi) (method described in [27]); α-tubulin is used as a loading control. **(B) **DNA isolated from micronuclei (2), anlagen (4) or macronuclei (6) was digested with BglII and separated using agarose gel electrophoresis and transferred to a nylon membrane; (1) is a DNA size marker. In Southern analyses using stad5 as a probe prominent signals in micronuclei (3) and anlagen (5), but not in macronuclei (7) are detected. Click here for file

Additional file 3 **Figure S3**. **Quantification of H3K9ac/K14ac and H3K27me3 during macronuclear development**. Image stacks were acquired with normalized microscopic settings. Subsequently the ratios of H3K9ac/K14ac and H3K27me3 to DNA were determined in microscopic sections using ImageJ to measure the mean signal intensities and the nuclear area covered by histone modification signals. For each developmental stage and signal between 20 and 60 measurements were made. **H3K9ac/K14ac: **No H3K9ac/K14ac is observed in micronuclei (m). However, the gain of H3K9ac/K14ac signals in early macronuclear anlagen (a_1_) leads to an increase of the H3K9ac/K14ac:DNA ratio in the diagram. During subsequent stages of polytenization this ratio does not change significantly, suggesting that *de novo *acetylation directly correlates with the growing DNA content during these first rounds of DNA amplification (stages a_2 _and a_3_). With the beginning of DNA elimination (e) the H3K9ac/K14ac:DNA ratio transiently increases, suggesting that DNA elimination primarily targets non-acetylated sequences. **H3K27me3: **H3K27me3 is abundant in micronuclei (m) as shown by an increased signal:DNA ratio. In early/mid macronuclear anlagen (a_1_/a_2_) the H3K27me3:DNA ratio decreases to a threshold level and increases between stages a_2 _and a_3 _during the first rounds of DNA amplification. These data confirm the microscopic observation that large-scale introduction of H3K27me3 occurs at advanced stages of macronuclear development. During DNA elimination stages (e) the H3K27me3:DNA ratio decreases, suggesting that the removal of this histone modification correlates with the elimination of DNA to reach a threshold level in mature macronuclei (M). Click here for file

Additional file 4 **Figure S4**. **ChIP analyses of H3S28p in macronuclear anlagen**. For real-time PCR analyses primers overlapping a scrambled and inverted part of the micronuclear actin I gene were selected to exclude contamination from the macronuclear actin I nanochromosome. Positions of PCR fragments for macronucleus destined ('actin I') and actin I flanking sequences ('flank_EF' and 'flank_GH') are marked above the diagram of the micronuclear actin I gene. Position of the PCR fragment 'stad5' is marked above the diagram of the micronucleus-specific sequence stad5. Click here for file

## References

[B1] Klose RJ, Zhang Y (2007). Regulation of histone methylation by demethylimination and demethylation. Nat Rev Mol Cell Biol.

[B2] Kouzarides T (2007). Chromatin modifications and their function. Cell.

[B3] Li B, Carey M, Workman JL (2007). The role of chromatin during transcription. Cell.

[B4] Martin C, Zhang Y (2005). The diverse functions of histone lysine methylation. Nat Rev Mol Cell Biol.

[B5] Shi Y, Lan F, Matson C, Mulligan P, Whetstine JR, Cole PA, Casero RA (2004). Histone demethylation mediated by the nuclear amine oxidase homolog LSD1. Cell.

[B6] Kim DH, Villeneuve LM, Morris KV, Rossi JJ (2006). Argonaute-1 directs siRNA-mediated transcriptional gene silencing in human cells. Nat Struct Mol Biol.

[B7] Mochizuki K, Fine NA, Fujisawa T, Gorovsky MA (2002). Analysis of a piwi-related gene implicates small RNAs in genome rearrangement in *Tetrahymena*. Cell.

[B8] Mochizuki K, Gorovsky MA (2004). Small RNAs in genome rearrangement in *Tetrahymena*. Curr Opin Genet Dev.

[B9] Mochizuki K, Gorovsky MA (2004). Conjugation-specific small RNAs in *Tetrahymena *have predicted properties of scan (scn) RNAs involved in genome rearrangement. Genes Dev.

[B10] Verdel A, Jia S, Gerber S, Sugiyama T, Gygi S, Grewal SI, Moazed D (2004). RNAi-mediated targeting of heterochromatin by the RITS complex. Science.

[B11] Zaratiegui M, Irvine DV, Martienssen RA (2007). Noncoding RNAs and gene silencing. Cell.

[B12] Prescott DM (1994). The DNA of ciliated protozoa. Microbiol Rev.

[B13] Ammermann D, Steinbruck G, Berger Lv, Hennig W (1974). The development of the macronucleus in the ciliated protozoan *Stylonychia mytilus*. Chromosoma.

[B14] Juranek SA, Lipps HJ (2007). New insights into the macronuclear development in ciliates. Int Rev Cytol.

[B15] Cavalcanti AR, Stover NA, Orecchia L, Doak TG, Landweber LF (2004). Coding properties of *Oxytricha trifallax *(*Sterkiella histriomuscorum*) macronuclear chromosomes: analysis of a pilot genome project. Chromosoma.

[B16] Meyer GF, Lipps HJ (1981). The formation of polytene chromosomes during macronuclear development of the hypotrichous ciliate *Stylonychia mytilus*. Chromosoma.

[B17] Meyer GF, Lipps HJ (1980). Chromatin elimination in the hypotrichous ciliate *Stylonychia mytilus*. Chromosoma.

[B18] Juranek SA, Rupprecht S, Postberg J, Lipps HJ (2005). snRNA and heterochromatin formation are involved in DNA excision during macronuclear development in stichotrichous ciliates. Eukaryot Cell.

[B19] Chalker DL, Yao MC (2001). Nongenic, bidirectional transcription precedes and may promote developmental DNA deletion in *Tetrahymena thermophila*. Genes Dev.

[B20] Mochizuki K, Gorovsky MA (2005). A Dicer-like protein in *Tetrahymena *has distinct functions in genome rearrangement, chromosome segregation, and meiotic prophase. Genes Dev.

[B21] Liu Y, Taverna SD, Muratore TL, Shabanowitz J, Hunt DF, Allis CD (2007). RNAi-dependent H3K27 methylation is required for heterochromatin formation and DNA elimination in *Tetrahymena*. Genes Dev.

[B22] Taverna SD, Coyne RS, Allis CD (2002). Methylation of histone H3 at lysine 9 targets programmed DNA elimination in *Tetrahymena*. Cell.

[B23] Aravin A, Gaidatzis D, Pfeffer S, Lagos-Quintana M, Landgraf P, Iovino N, Morris P, Brownstein MJ, Kuramochi-Miyagawa S, Nakano T (2006). A novel class of small RNAs bind to MILI protein in mouse testes. Nature.

[B24] Girard A, Sachidanandam R, Hannon GJ, Carmell MA (2006). A germline-specific class of small RNAs binds mammalian Piwi proteins. Nature.

[B25] Fetzer CP, Hogan DJ, Lipps HJ (2002). A PIWI homolog is one of the proteins expressed exclusively during macronuclear development in the ciliate *Stylonychia lemnae*. Nucleic Acids Res.

[B26] Paschka AG, Horejschi V, Jonsson F, Lindecke A, Weier G, Kaltschmidt C, Lipps HJ (2005). A microarray analysis of developmentally regulated genes during macronuclear differentiation in the stichotrichous ciliate *Stylonychia lemnae*. Gene.

[B27] Paschka AG, Jönsson F, Maier V, Möllenbeck M, Paeschke K, Postberg J, Rupprecht S, Lipps HJ (2003). The use of RNAi to analyze gene function in spirotrichous ciliates. European Journal of Protistology.

[B28] Duharcourt S, Yao MC (2002). Role of histone deacetylation in developmentally programmed DNA rearrangements in *Tetrahymena thermophila*. Eukaryot Cell.

[B29] Garcia BA, Hake SB, Diaz RL, Kauer M, Morris SA, Recht J, Shabanowitz J, Mishra N, Strahl BD, Allis CD, Hunt DF (2007). Organismal differences in post-translational modifications in histones H3 and H4. J Biol Chem.

[B30] Garnier O, Serrano V, Duharcourt S, Meyer E (2004). RNA-mediated programming of developmental genome rearrangements in *Paramecium tetraurelia*. Mol Cell Biol.

[B31] Liu Y, Mochizuki K, Gorovsky MA (2004). Histone H3 lysine 9 methylation is required for DNA elimination in developing macronuclei in *Tetrahymena*. Proc Natl Acad Sci USA.

[B32] Taverna SD, Ueberheide BM, Liu Y, Tackett AJ, Diaz RL, Shabanowitz J, Chait BT, Hunt DF, Allis CD (2007). Long-distance combinatorial linkage between methylation and acetylation on histone H3 N termini. Proc Natl Acad Sci USA.

[B33] Morris SA, Rao B, Garcia BA, Hake SB, Diaz RL, Shabanowitz J, Hunt DF, Allis CD, Lieb JD, Strahl BD (2007). Identification of histone H3 lysine 36 acetylation as a highly conserved histone modification. J Biol Chem.

[B34] Ruthenburg AJ, Allis CD, Wysocka J (2007). Methylation of lysine 4 on histone H3: intricacy of writing and reading a single epigenetic mark. Mol Cell.

[B35] van Dijk K, Marley KE, Jeong BR, Xu J, Hesson J, Cerny RL, Waterborg JH, Cerutti H (2005). Monomethyl histone H3 lysine 4 as an epigenetic mark for silenced euchromatin in *Chlamydomonas*. Plant Cell.

[B36] Ng HH, Robert F, Young RA, Struhl K (2003). Targeted recruitment of Set1 histone methylase by elongating Pol II provides a localized mark and memory of recent transcriptional activity. Mol Cell.

[B37] Santos-Rosa H, Schneider R, Bannister AJ, Sherriff J, Bernstein BE, Emre NC, Schreiber SL, Mellor J, Kouzarides T (2002). Active genes are tri-methylated at K4 of histone H3. Nature.

[B38] Schneider R, Bannister AJ, Myers FA, Thorne AW, Crane-Robinson C, Kouzarides T (2004). Histone H3 lysine 4 methylation patterns in higher eukaryotic genes. Nat Cell Biol.

[B39] Guenther MG, Levine SS, Boyer LA, Jaenisch R, Young RA (2007). A chromatin landmark and transcription initiation at most promoters in human cells. Cell.

[B40] Postberg J, Alexandrova O, Cremer T, Lipps HJ (2005). Exploiting nuclear duality of ciliates to analyse topological requirements for DNA replication and transcription. J Cell Sci.

[B41] Postberg J, Alexandrova O, Lipps HJ (2006). Synthesis of pre-rRNA and mRNA is directed to a chromatin-poor compartment in the macronucleus of the spirotrichous ciliate *Stylonychia lemnae*. Chromosome Res.

[B42] Lee JS, Shilatifard A (2007). A site to remember: H3K36 methylation a mark for histone deacetylation. Mutat Res.

[B43] Bernhard D (1999). Several highly divergent histone H3 genes are present in the hypotrichous ciliate *Stylonychia lemnae*. FEMS Microbiol Lett.

[B44] Peters AH, Kubicek S, Mechtler K, O'Sullivan RJ, Derijck AA, Perez-Burgos L, Kohlmaier A, Opravil S, Tachibana M, Shinkai Y, Martens JH, Jenuwein T (2003). Partitioning and plasticity of repressive histone methylation states in mammalian chromatin. Mol Cell.

[B45] Cao R, Zhang Y (2004). The functions of E(Z)/EZH2-mediated methylation of lysine 27 in histone H3. Curr Opin Genet Dev.

[B46] Schlegel M, Muller S, Ruder F, Büsen W (1990). Transcriptionally inactive micronuclei, macronuclear anlagen and transcriptionally active macronuclei differ in histone composition in the hypotrichous ciliate *Stylonychia lemnae*. Chromosoma.

[B47] Goto H, Yasui Y, Nigg EA, Inagaki M (2002). Aurora-B phosphorylates Histone H3 at serine28 with regard to the mitotic chromosome condensation. Genes Cells.

[B48] Murnion ME, Adams RR, Callister DM, Allis CD, Earnshaw WC, Swedlow JR (2001). Chromatin-associated protein phosphatase 1 regulates aurora-B and histone H3 phosphorylation. J Biol Chem.

[B49] Mateescu B, England P, Halgand F, Yaniv M, Muchardt C (2004). Tethering of HP1 proteins to chromatin is relieved by phosphoacetylation of histone H3. EMBO Rep.

[B50] Fischle W, Tseng BS, Dormann HL, Ueberheide BM, Garcia BA, Shabanowitz J, Hunt DF, Funabiki H, Allis CD (2005). Regulation of HP1-chromatin binding by histone H3 methylation and phosphorylation. Nature.

[B51] Daujat S, Zeissler U, Waldmann T, Happel N, Schneider R (2005). HP1 binds specifically to Lys26-methylated histone H1.4, whereas simultaneous Ser27 phosphorylation blocks HP1 binding. J Biol Chem.

[B52] Fischle W, Wang Y, Allis CD (2003). Binary switches and modification cassettes in histone biology and beyond. Nature.

[B53] Sun JM, Chen HY, Espino PS, Davie JR (2007). Phosphorylated serine 28 of histone H3 is associated with destabilized nucleosomes in transcribed chromatin. Nucleic Acids Res.

[B54] Mollenbeck M, Cavalcanti AR, Jonsson F, Lipps HJ, Landweber LF (2006). Interconversion of germline-limited and somatic DNA in a scrambled gene. J Mol Evol.

[B55] Madireddi MT, Coyne RS, Smothers JF, Mickey KM, Yao MC, Allis CD (1996). Pdd1p, a novel chromodomain-containing protein, links heterochromatin assembly and DNA elimination in *Tetrahymena*. Cell.

[B56] Maercker C, Kortwig H, Nikiforov MA, Allis CD, Lipps HJ (1999). A nuclear protein involved in apoptotic-like DNA degradation in *Stylonychia*: implications for similar mechanisms in differentiating and starved cells. Mol Biol Cell.

[B57] Allis CD, Berger SL, Cote J, Dent S, Jenuwien T, Kouzarides T, Pillus L, Reinberg D, Shi Y, Shiekhattar R, Shilatifard A, Workman J, Zhang Y (2007). New nomenclature for chromatin-modifying enzymes. Cell.

[B58] Bannister AJ, Zegerman P, Partridge JF, Miska EA, Thomas JO, Allshire RC, Kouzarides T (2001). Selective recognition of methylated lysine 9 on histone H3 by the HP1 chromo domain. Nature.

[B59] Lachner M, O'Carroll D, Rea S, Mechtler K, Jenuwein T (2001). Methylation of histone H3 lysine 9 creates a binding site for HP1 proteins. Nature.

[B60] Nakayama J, Rice JC, Strahl BD, Allis CD, Grewal SI (2001). Role of histone H3 lysine 9 methylation in epigenetic control of heterochromatin assembly. Science.

[B61] Meshorer E, Misteli T (2006). Chromatin in pluripotent embryonic stem cells and differentiation. Nat Rev Mol Cell Biol.

[B62] Francastel C, Schubeler D, Martin DI, Groudine M (2000). Nuclear compartmentalization and gene activity. Nat Rev Mol Cell Biol.

[B63] Arney KL, Fisher AG (2004). Epigenetic aspects of differentiation. J Cell Sci.

[B64] Meshorer E, Yellajoshula D, George E, Scambler PJ, Brown DT, Misteli T (2006). Hyperdynamic plasticity of chromatin proteins in pluripotent embryonic stem cells. Dev Cell.

[B65] Santos F, Hendrich B, Reik W, Dean W (2002). Dynamic reprogramming of DNA methylation in the early mouse embryo. Dev Biol.

[B66] Erhardt S, Su IH, Schneider R, Barton S, Bannister AJ, Perez-Burgos L, Jenuwein T, Kouzarides T, Tarakhovsky A, Surani MA (2003). Consequences of the depletion of zygotic and embryonic enhancer of zeste 2 during preimplantation mouse development. Development.

[B67] Cammas F, Oulad-Abdelghani M, Vonesch JL, Huss-Garcia Y, Chambon P, Losson R (2002). Cell differentiation induces TIF1beta association with centromeric heterochromatin via an HP1 interaction. J Cell Sci.

[B68] Brower-Toland B, Findley SD, Jiang L, Liu L, Yin H, Dus M, Zhou P, Elgin SC, Lin H (2007). *Drosophila *PIWI associates with chromatin and interacts directly with HP1a. Genes Dev.

[B69] Laemmli UK (1970). Cleavage of structural proteins during the assembly of the head of bacteriophage T4. Nature.

[B70] Zinner R, Albiez H, Walter J, Peters AH, Cremer T, Cremer M (2006). Histone lysine methylation patterns in human cell types are arranged in distinct three-dimensional nuclear zones. Histochem Cell Biol.

