# Supplementary figures and images for: Spatial and temporal plasticity of chromatin during programmed DNA-reorganization in Stylonychia macronuclear development
Source: Epigenetics Chromatin. 2008 Oct 27;1:3. doi: 10.1186/1756-8935-1-3 (PMC2603335; doi:10.1186/1756-8935-1-3)

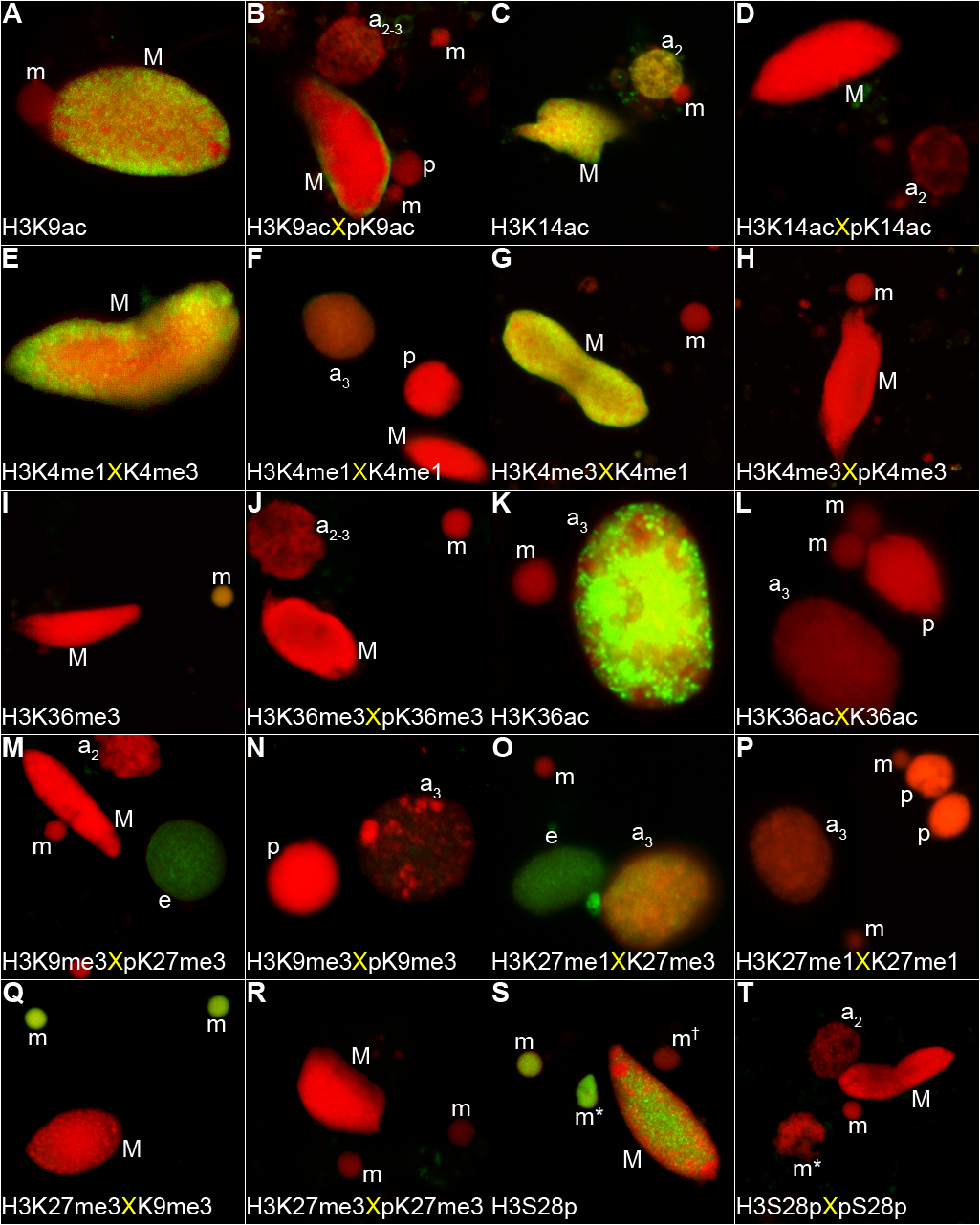

Supplement: Additional file 1 — Figure S1. Peptide competition assays. Immunofluorescence microscopic analyses show the specificity of the antibodies used in this study. Grey-scale images were acquired using a Leica DM RB transmission light microscope equipped with a Leica DC300F CCD camera; subsequently false colors were assigned to each channel. Green: post-translational histone modifications. DAPI was used for DNA counterstaining (red). (A) H3K9ac antibody (ab); (B) H3K9ac ab competed with the immunizing peptide; (C) H3K14ac ab; (D) H3K14ac ab competed with the immunizing peptide; (E) H3K4me1 ab competed with a peptide trimethylated at K4; (F) H3K4me1 ab competed with the immunizing peptide; (G) H3K4me3 ab competed with a peptide monomethylated at K4; (H) H3K4me3 ab competed with the immunizing peptide; (I) H3K36me3 ab; (J) H3K36me3 ab competed with the immunizing peptide; (K) H3K36ac ab; (L) H3K36ac ab competed with the immunizing peptide; (M) H3K9me3 ab competed with a peptide trimethylated at K27; (N) H3K9me3 ab competed with the immunizing peptide; (O) H3K27me1 ab competed with a peptide trimethylated at K27; (P) H3K27me1 ab competed with the immunizing peptide; (Q) H3K27me3 ab competed with a peptide trimethylated at K9; (R) H3K27me3 ab competed with the immunizing peptide; (S) H3S28p ab; (T) H3S28p ab competed with the immunizing peptide. [file 1756-8935-1-3-S1.png]

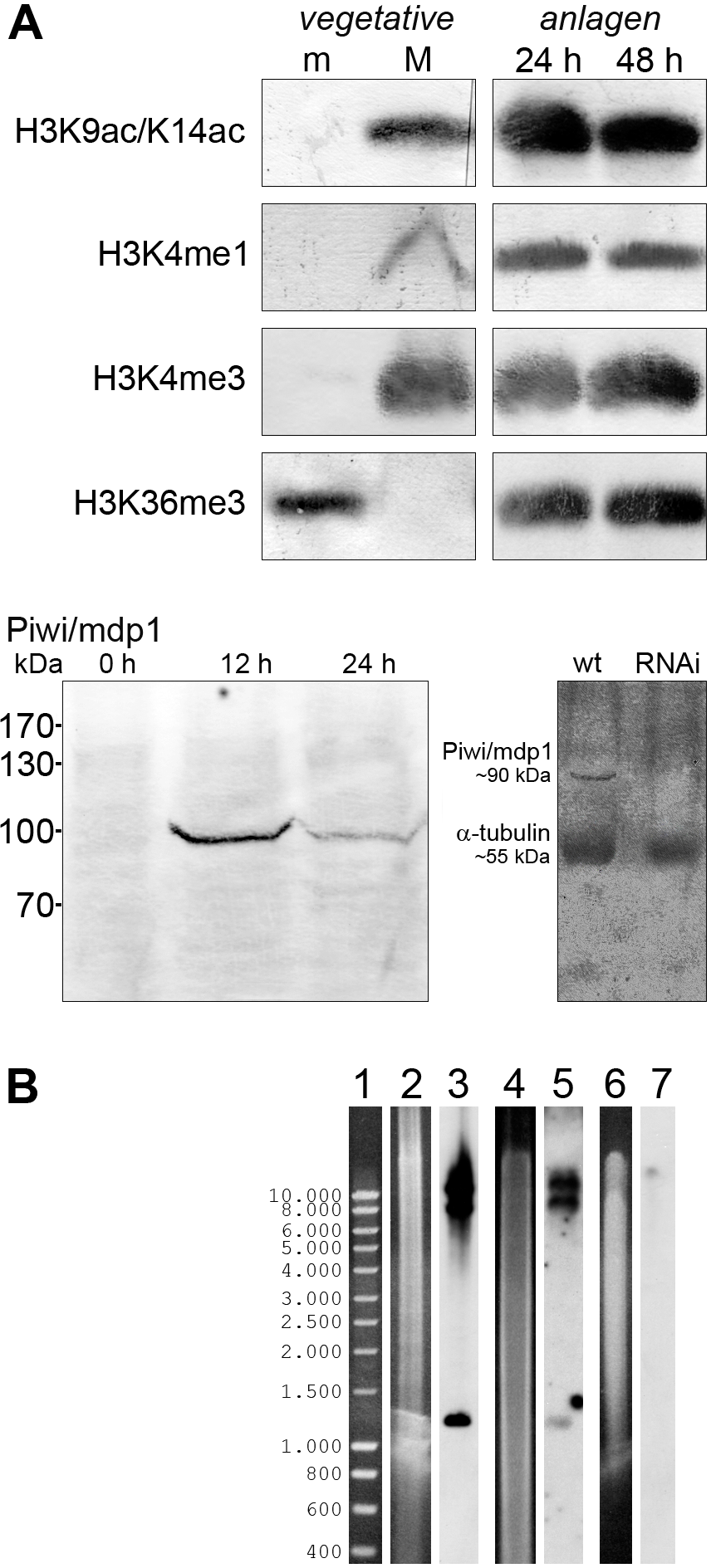

Supplement: Additional file 2 — Figure S2. (A) Western analyses confirm the occurrence of post-translational histone H3 modifications in micronuclei (m) and macronuclei (M) as well as in anlagen 24 h and 48 h post conjugation. Piwi/mdp1 (Western blot, left) is not detected in vegetative cell shortly before conjugation (0 h). A < 100 kDa protein is detected during macronuclear development (12 h and 24 h post conjugation) using an antibody targeted to a Piwi family protein. Western blot, right: The band detected with the Piwi antibody (lane wt) becomes weaker or disappears completely upon RNAi treatment (lane RNAi) (method described in [27]); α-tubulin is used as a loading control. (B) DNA isolated from micronuclei (2), anlagen (4) or macronuclei (6) was digested with BglII and separated using agarose gel electrophoresis and transferred to a nylon membrane; (1) is a DNA size marker. In Southern analyses using stad5 as a probe prominent signals in micronuclei (3) and anlagen (5), but not in macronuclei (7) are detected. [file 1756-8935-1-3-S2.png]

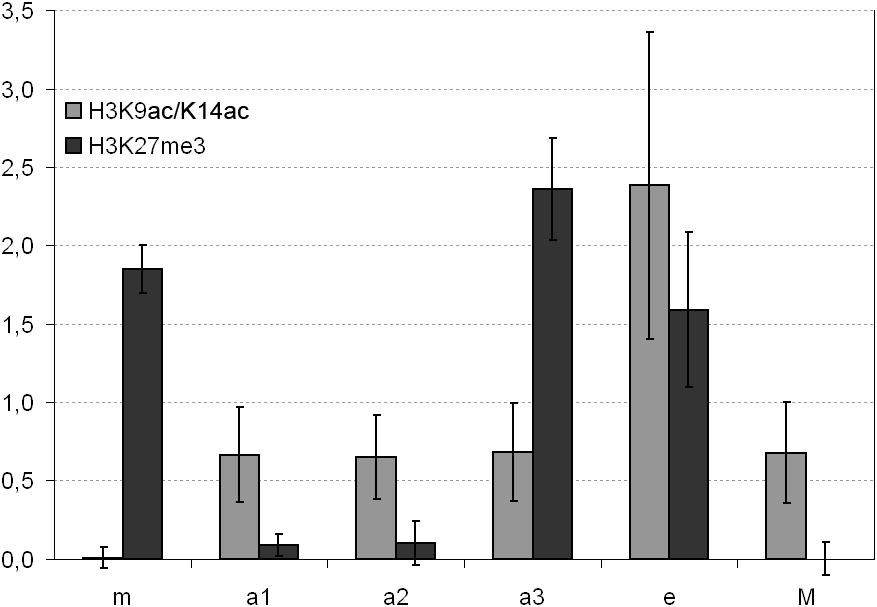

Supplement: Additional file 3 — Figure S3. Quantification of H3K9ac/K14ac and H3K27me3 during macronuclear development. Image stacks were acquired with normalized microscopic settings. Subsequently the ratios of H3K9ac/K14ac and H3K27me3 to DNA were determined in microscopic sections using ImageJ to measure the mean signal intensities and the nuclear area covered by histone modification signals. For each developmental stage and signal between 20 and 60 measurements were made. H3K9ac/K14ac: No H3K9ac/K14ac is observed in micronuclei (m). However, the gain of H3K9ac/K14ac signals in early macronuclear anlagen (a1) leads to an increase of the H3K9ac/K14ac:DNA ratio in the diagram. During subsequent stages of polytenization this ratio does not change significantly, suggesting that de novo acetylation directly correlates with the growing DNA content during these first rounds of DNA amplification (stages a2 and a3). With the beginning of DNA elimination (e) the H3K9ac/K14ac:DNA ratio transiently increases, suggesting that DNA elimination primarily targets non-acetylated sequences. H3K27me3: H3K27me3 is abundant in micronuclei (m) as shown by an increased signal:DNA ratio. In early/mid macronuclear anlagen (a1/a2) the H3K27me3:DNA ratio decreases to a threshold level and increases between stages a2 and a3 during the first rounds of DNA amplification. These data confirm the microscopic observation that large-scale introduction of H3K27me3 occurs at advanced stages of macronuclear development. During DNA elimination stages (e) the H3K27me3:DNA ratio decreases, suggesting that the removal of this histone modification correlates with the elimination of DNA to reach a threshold level in mature macronuclei (M). [file 1756-8935-1-3-S3.png]

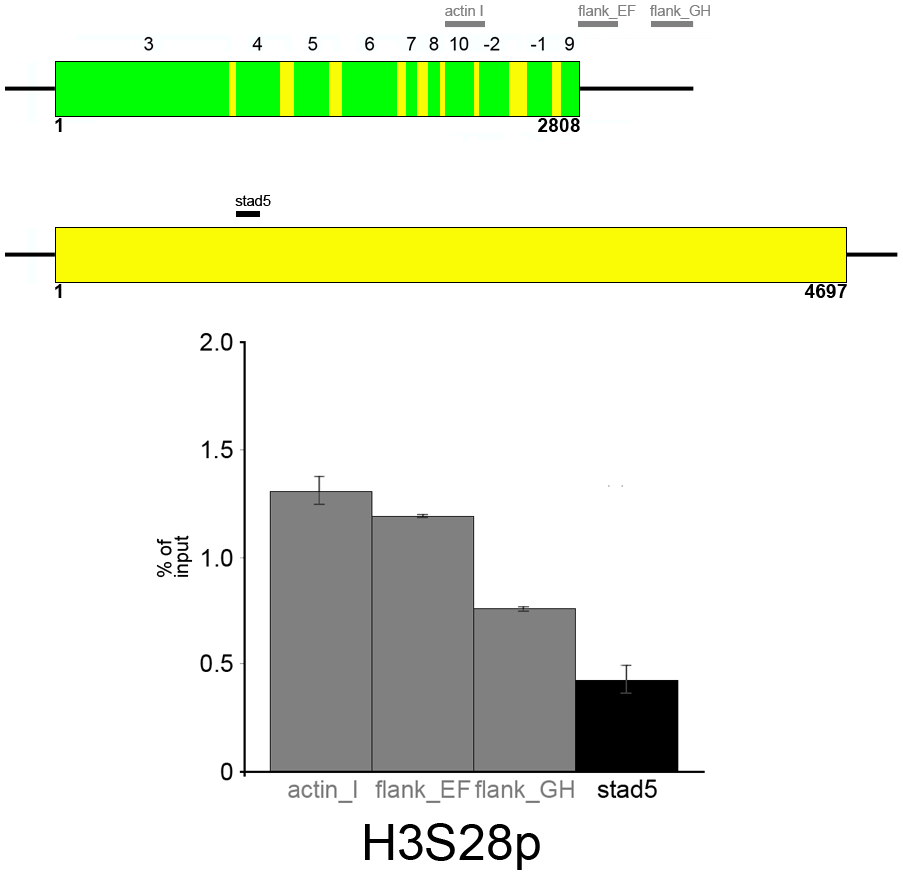

Supplement: Additional file 4 — Figure S4. ChIP analyses of H3S28p in macronuclear anlagen. For real-time PCR analyses primers overlapping a scrambled and inverted part of the micronuclear actin I gene were selected to exclude contamination from the macronuclear actin I nanochromosome. Positions of PCR fragments for macronucleus destined ('actin I') and actin I flanking sequences ('flank_EF' and 'flank_GH') are marked above the diagram of the micronuclear actin I gene. Position of the PCR fragment 'stad5' is marked above the diagram of the micronucleus-specific sequence stad5. [file 1756-8935-1-3-S4.png]
